# Low-fat and whole milk consumption in relation to cardiovascular disease–related and all-cause mortality: a prospective cohort study in 3 Norwegian counties

**DOI:** 10.1016/j.ajcnut.2025.07.035

**Published:** 2025-08-07

**Authors:** Erik Kristoffer Arnesen, Jacob Juel Christensen, Ida Laake, Monica H Carlsen, Marit B Veierød, Kjetil Retterstøl

**Affiliations:** 1 Department of Nutrition, Institute of Basic Medical Sciences, University of Oslo, Oslo, Norway; 2 Norwegian Institute of Public Health, Oslo, Norway; 3 Oslo Centre for Biostatistics and Epidemiology, Department of Biostatistics, Institute of Basic Medical Sciences, University of Oslo Faculty of Medicine, Oslo, Norway; 4 The Lipid Clinic, Department of Endocrinology, Morbid Obesity and Preventive Medicine, Oslo University Hospital, Oslo, Norway

**Keywords:** milk, dairy products, cardiovascular disease, mortality, cohort study

## Abstract

**Background:**

The impact of milk consumption on cardiovascular disease (CVD) risk or mortality is unclear, and specific comparisons between types of milk are scarce.

**Objectives:**

We aimed to investigate the associations between milk consumption, measured in a large Norwegian cohort in 3 counties during 1974–1988, and CVD–related mortality and all-cause mortality and to compare the associations between whole (full-fat) and low-fat milk.

**Methods:**

This population-based, prospective cohort study included adults invited to repeated health screenings in the Norwegian Counties Study (>80% attendance). Dietary intake was assessed from food frequency questionnaires at each screening in 1974–1988. Cox regression analysis was used to estimate hazard ratios (HRs) with 95% CIs for the associations between milk intake (assessed as cumulative mean intake from repeated questionnaires) and risk of death from all CVD, ischemic heart disease (IHD), acute myocardial infarction (AMI), and all causes.

**Results:**

Among 73,860 participants included in this study (mean age: 41.2 years at baseline; 49% females), 26,393 deaths occurred (8590 from CVD, 4372 from IHD, and 3047 from AMI) during a median follow-up of 33 y. Comparing the highest with the lowest quintile of overall milk intake, the HR was 1.12 (95% CI: 1.03, 1.21) for CVD-related mortality and 1.22 (95% CI: 1.16, 1.28) for all-cause mortality. For whole milk, high compared with no intake was positively associated with CVD (HR: 1.07; 95% CI: 0.99, 1.16), IHD (HR: 1.13; 95% CI: 1.01, 1.27), and all-cause mortality (HR: 1.15; 95% CI: 1.10, 1.21). Compared with whole milk, low-fat milk intake was associated with lower CVD-related, IHD-related, AMI-related, and all-cause mortality. The associations were attenuated in analyses considering only baseline intakes.

**Conclusions:**

In this cohort with high milk consumption, whole milk is associated with higher CVD and all-cause mortality, whereas low-fat milk is not. Low-fat milk may be associated with lower risks than whole milk.

## Introduction

Dietary guidelines worldwide often recommend consuming low-fat or fat-free dairy products over full-fat alternatives partly due to concerns about SFAs in dairy products [1,2]. Dairy products are among the primary sources of SFA, which are positively associated with cardiovascular disease (CVD)-related and all-cause mortality [3]. Nonetheless, the evidence linking full-fat dairy directly to increased CVD risk remains limited [4]. It has been proposed that SFA from dairy products are less harmful than SFA from other sources, as some studies have shown inverse associations between cheese intake and CVD or cardiometabolic risk factors [[5], [6], [7], [8], [9]]. This highlights the need to explore the health effects associated with various subtypes of dairy products in greater detail [6].

In systematic reviews of 20–30 prospective cohort studies, intake of overall dairy products has not been associated with risk of CVD or coronary artery disease (CAD) [6,10,11]. However, a meta-analysis by Naghshi et al. [12] found that high-fat (whole) milk intake, but not overall milk, was associated with all-cause and CVD-related mortality [12]. Specific comparisons between types of milk are scarce. Kiesswetter et al. [13] recently investigated the potential impact of different dairy product substitutions on risk of all-cause death and cardiometabolic disease in a systematic review and meta-analysis. However, an insufficient number of studies precluded a meta-analysis comparing whole and low-fat milk.

Norway is traditionally a high milk-consuming country. The consumption of whole milk increased during the first half of the 1900s, reaching 194 L per capita in 1953, at the expense of skim milk [14]. The promotion of milk as “health in every drop” was challenged when the high consumption of fat was implicated in the sharp increase in ischemic heart disease (IHD) after World War II [15]. Since the 1970s, a shift from whole to low-fat milk became a political aim. By 1989, the consumption of whole milk had declined to 64 L per capita, a change particularly driven by the introduction of semiskim milk (1.5% fat) [16]. Recent trends, however, suggest a resurgence in the popularity of whole milk and high-fat dairy products [17,18].

Given these developments and research gaps, we aimed to evaluate the associations between milk intake (overall intake and comparing low-fat with whole milk) and deaths from CVD [including IHD, and acute myocardial infarction (AMI)] and all causes within a Norwegian cohort studied during the 1970s and 1980s, to examine the potential health consequences of changing milk consumption patterns during that historical period. As we previously observed a positive association between intake of ruminant *trans*-fatty acids (from dairy fat and red meat) and sudden deaths in this cohort, we also considered sudden death of unknown cause as a secondary outcome [19].

## Methods

### Study population

The Norwegian Counties Study is a population-based, prospective cohort study conducted by the National Health Screening Service in 3 counties in Norway: Finnmark, Sogn and Fjordane, and Oppland, located in the north, west, and east (inland) regions of Norway, respectively. The counties were predominantly rural areas with varying socioeconomic profiles. They were selected partly for their contrasting CVD-related mortality rates, which were higher in Finnmark and lower in the other counties compared with Norway as a whole [20]. In each county, 3 cardiovascular health screenings were performed between 1974 and 1988 [20]. At screening I (1974–1976), all residents aged 35–49 y and a 10% random sample of residents aged 20–34 y were invited, and 65,624 individuals (88%) attended [21]. The same individuals, along with random samples of younger and older birth cohorts, were invited to subsequent screenings 3–5 y later: screening II (1977–1983, 87.5% attendance) and screening III (1985–1988, 83.6% attendance).

Ethical approval was obtained from the Regional Committee for Medical Research Ethics Norway (no. 2018/1302), the Norwegian Institute of Public Health and the University of Oslo’s internal privacy protection commission.

### Dietary assessments

At each screening visit, participants received a short semiquantitative food frequency questionnaire (FFQ), developed by the Section for Dietary Research, University of Oslo. In Finnmark county, the FFQ was only administered in 1 municipality at screening I. The FFQs remained largely identical across screenings and included ∼70 questions about foods, with a particular emphasis on usually consumed “everyday” foods and meals that were important sources of fat and contributed significantly to daily energy intake. Participants reported the number of glasses of milk (any type) typically consumed per day (ranging from do not drink/drink <1 to ≥6). Based on the number of glasses per day, we estimated daily overall milk intake using a standard portion size of 150 g per glass (<1 glass was set to 2 glasses per week and ≥6 glasses was set to 6.5 glasses/d to calculate grams per day) [22]. A separate question about type of milk had 5 mutually exclusive categories of milk: whole (full-fat) milk (3.8% fat), skim milk (0.1% fat), semiskim milk (1.5% fat), mixed whole milk and skim milk, or do not drink milk. Because the use of semiskim milk was virtually nonexistent before screening III, we combined skim and semiskim/low-fat milk into 1 category, termed low-fat, in our analyses. We defined daily intake of each type of milk (grams per day) based on the reported number of glasses of overall milk, as described earlier. If only the type, but not amount, of milk, was reported, the lowest quantity was assigned. Respondents who answered do not drink milk or who did not respond to any of the milk questions were categorized as nonconsumers (0 glasses/d) [23]. For participants reporting to mix whole and skim milk, the total amount was divided equally between whole and low-fat milk, a common recommendation before semiskim milk became available [24,25], to quantify the intake for the separate analyses by milk type. Milk as an ingredient in foods or dishes (e.g., sauces or fish products) was not included in the milk quantification but was included in energy and nutrient calculations. From the FFQ, we also identified other sources of SFA [red/processed meat, cheese, and type and amount of fat (butter, margarine, or no fat) used on bread or meat/fish dishes], as well as intake of coffee (cups/day), and sweetened soft drinks (glasses/week).

Energy (not including alcohol) intake (kilojoules per day) and nutrient intakes were estimated with official Norwegian food composition tables from 1984 and 1991 [26,27] for participants with <10 unanswered items in the FFQ (93% of the FFQ responders at screening I, 82% in screening II, and 84% in screening III). Nutrient densities were calculated as percentage of energy (E%) for macronutrients and grams per 1000 kcal for micronutrients. Participants with energy intake considered implausible (<1195 or >16,736 kJ/d) were excluded from the analyses.

The FFQ was compared with a single 24-h recall (24HR) interview among a subset of participants at screening I (*n* = 1609) [28]. Proportions of whole and skim milk consumers (among milk consumers) were similar with the 2 methods. Most (80% of the males and 74% of the females) of those who reported whole milk in the FFQ also did so in the 24HR.

### Covariate assessments

A comprehensive questionnaire concerning lifestyle habits, health, and civil status was included with the invitation letter and brought by the participants to the study site, where trained personnel checked the filled-in questionnaire and conducted clinical examinations.

In the questionnaire, participants reported their recreational physical activity (sedentary, moderate, high, and vigorous) and occupational physical activity (mostly sedentary, walking, walking and lifting, and heavy manual labor) during the past year [29]. Smoking status was categorized as never, previous, or current smoking, and smoking duration as years of daily smoking. A history of CVD, diabetes, and use of antihypertensive medications and/or nitroglycerine, hereafter termed comorbidities, was self-reported. Marital status (unmarried, married, widowed, divorced, or separated) was also self-reported, whereas attained education at the time of each screening was obtained through linkage with Statistics Norway and categorized as compulsory (1–9 y), upper secondary (10–12 y), and higher (≥13 y) education.

Weight and height were measured at the physical examinations, and BMI was calculated (in kg/m^2^). Blood samples were analyzed for nonfasting serum total cholesterol, HDL cholesterol, and triglycerides at the central laboratory of Ullevaal University Hospital, Oslo, Norway, for all screenings in all counties [30]. HDL cholesterol was only available from screenings II and III. We calculated non-HDL cholesterol, a proxy for atherogenic lipoproteins, by subtracting HDL cholesterol from total cholesterol. For participants with total cholesterol, HDL cholesterol, and triglycerides, we further calculated LDL cholesterol using the Martin/Hopkins equations if triglyceride concentrations were <400 mg/dL (∼4.5 mmol/L) [31].

### Mortality outcomes and follow-up

The primary outcomes were deaths due to CVD (i.e., diseases of the circulatory system, including IHD), IHD (AMI, angina pectoris, and chronic IHD), and AMI alone, as well as deaths from all causes. Causes of death were obtained from the national Cause of Death Registry using each participant’s personal identification number. The causes were defined using the International Classification of Diseases (ICD) codes: CVD (ICD8: 390–458; ICD9: 390–459; ICD10: I00–I99), IHD (ICD8–9: 410–414, 4140, 4143, 4148–4149; ICD10: I20–I25), and AMI (ICD8–9: 410–411; ICD10: I21–I22), in accordance with the European Shortlist for Causes of Death (COD-SL-2012). ICD8: 782.4, 795; ICD9: 798.1, 798.2; and ICD10: R96 were considered for the secondary outcome of sudden deaths.

The participants’ first screening with age ≥18 y and <65 y and available dietary data as well as information on covariates as specified earlier was defined as baseline. Person-years of follow-up were calculated from baseline until date of death, emigration, or until 31 December, 2018, whichever came first. We censored for death of other causes in the analyses of disease-specific deaths.

### Statistical analysis

Intake of overall, whole, and low-fat milk was energy adjusted using the residual method [32]. To account for long-term intake and changes in intake over time, we calculated cumulative mean intakes of milk using milk intake at screening I until screening II, the mean intake at screenings I and II from screening II until screening III, and so forth [33]. If only intakes at baseline were available, they were carried forward for subsequent periods. Overall milk intake was categorized into quintiles of cumulative mean intake across the total study sample. Considering the large proportions of nonconsumers of the specific milk types, intake amounts for types of milk were categorized into quartiles of cumulative mean intake, with nonconsumers of each type of milk included as a separate category, following methods from previous studies of milk types [[34], [35], [36], [37]]. This category comprises both individuals reporting no milk intake and those who consumed other types of milk but not the type under analysis.

We used Cox regression models to estimate hazard ratios (HRs) and 95% CIs as follows: *1*) across quintiles of cumulative mean overall milk intake, calculated from repeated dietary assessments; *2*) for types of milk, with whole milk as the reference; and *3*) across quartiles of cumulative mean whole and low-fat milk intake with nonconsumers of the respective type of milk as reference. The latter thus estimates risk associated with whole or low-fat milk compared with not consuming that type of milk or any milk. The models were stratified by birth cohort (<1930, 1930–1934, 1935–1939, 1940–1944, 1945–1949, 1950–1954, and ≥1955) and age was used as the time scale, starting from each participant’s age at baseline, as defined earlier. Milk intake was also analyzed as a continuous variable to assess linear trends, with results reported per 100-g/d increment. Tests of interaction between milk intake and follow-up time indicated no violations of the proportional hazards assumption [38].

Model 1 included age (time scale), sex, and cumulative mean energy intake (continuous, calculated as described earlier for milk intake). In model 2, we further adjusted for potentially confounding variables informed by subject-matter knowledge regarding milk consumption patterns in Norway [39] and established associations with mortality or CVD risk. In addition to the variables in model 1, we included BMI (continuous), recreational and occupational physical activity, smoking status, attained education, marital status, and self-reported comorbidities at the time of each screening. As we have previously found associations between intake of SFA and all-cause and CVD-related mortality in this cohort, model 2 also included intake of the other dietary sources of SFA as described above [40]. Additionally, model 2 included adjustment for intake of coffee and sweetened soft drinks because a higher intake of milk might lead to a lower intake of coffee and soft drinks or vice versa. Intakes of dietary fiber (E%) and vitamin C (mg/1000 kcal) were also included in model 2. To model the replacement of whole milk with low-fat milk, we included both milk types in the model and calculated the differences in their respective regression coefficients, variances, and covariances to estimate HRs and 95% CIs. This approach can accurately estimate substitution effects for between 2 components when measured in consistent units [41,42]. The analysis of overall milk intake was also adjusted for type of milk in a separate model (model 3), whereas the categorical analysis comparing types of milk was adjusted for quantity of overall milk intake to estimate associations for each type of milk assuming a constant overall milk intake. All covariates, except sex, were updated at each attended screening where information was available. Because the proportion of missing covariate data was low, we performed complete case analysis.

Motivated by previous studies on SFA and ruminant *trans*-fatty acids in this cohort indicating sex-specific and BMI-specific associations with mortality [19,40], we performed stratified analyses to explore potential effect modification by these characteristics. We compared models with and without crossproduct terms to test for multiplicative interaction using the likelihood ratio test. Statistical analyses were performed using Stata software 18.0.

We examined the associations between intake of each type of milk and total, non-HDL cholesterol, and LDL cholesterol using multivariable mixed-effects models, with unstructured covariance structure, to account for repeated measures, with participant and screening as random effects. We adjusted for age and the covariates in model 2 as well as overall milk intake.

Several sensitivity analyses were performed. First, the associations between milk intake (overall and for each type of milk) and mortality were examined using glasses per day (<1, 1, 2, 3, or ≥4 glasses/d) as unit of analysis. To examine the influence of the repeated dietary assessments in minimizing exposure misclassification, we repeated the main analyses using only baseline milk intake data (collected at the participant’s first screening meeting the baseline criteria) instead of cumulative mean intakes. Furthermore, to address potential reverse causation bias, we excluded the first 5 y of follow-up. Additionally, we excluded participants who reported the presence of comorbidities or who reported to be on a special diet, as these conditions could introduce bias through reverse causation. We also excluded nonmilk consumers in the reference groups in sensitivity analyses of milk types because these participants might have abstained from drinking milk due to pre-existing disease. Because the choice of specific milk types might have been related to other dietary choices, we further adjusted for the intake of other foods that could be associated with choice of milk and the outcomes, including bread, bread toppings (cold cuts, fish, liver paste, jam, nut or sweet spreads), and eggs in another sensitivity analysis. Intake of beer, wine, and/or liquor was also included in this analysis.

## Results

**FIGURE 1:**
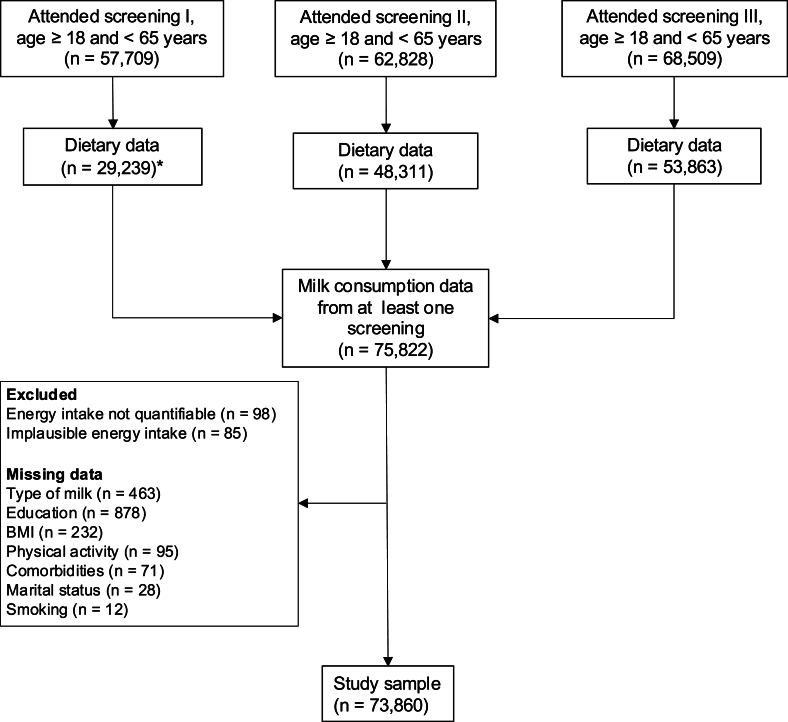
Flow-chart of participants, Norwegian Counties Study. Of the total 73,860 participants, 34,178 (46.3%) had 1 diet assessment, 23,699 (32.1%) had 2 diet assessments, and 15,983 (21.6%) had 3 diet assessments. ∗Food frequency questionnaires were not distributed in all municipalities in screening I.

Of 91,856 participants aged 18–64 y at baseline, dietary data, including number of glasses of milk, was available for 75,822 participants. After excluding participants with missing or implausible energy intake data (*n* = 183) and missing information on type of milk (*n* = 463) and covariates *(*n = 1316), the final study sample included 73,860 subjects (Figure 1). Mean (SD) age at baseline was 41.2 (7.4) y, and 50.9% of the participants were males (Table 1) [29].

**TABLE 1:** Baseline characteristics according to quintiles of overall milk intake (g/d) at baseline in the Norwegian Counties Study (*N* = 73,860).

	Quintile of overall milk intake					Total
	Q1	Q2	Q3	Q4	Q5	
*n*	14,738	14,836	14,852	14,618	14,816	73,860
Overall milk intake (g/d), energy adjusted)	106 (63)	218 (25)	322 (33)	431 (34)	628 (123)	341 (192)
Milk type						
Whole (full-fat, 3.8% fat)	52.5	58.5	61	54.8	49.8	55.3
Mixed whole/skimmed	8.5	13.2	15.3	15.2	15.9	13.6
Low-fat (≤1.5% fat)	14.5	28.3	23.7	30	34.3	26.2
Do not drink milk	24.5	0	0	0	0	0
Age (y)	42.0 (6.8)	42.0 (7.0)	41.5 (7.1)	40.8 (7.5)	39.6 (8.2)	41.2 (7.4)
Sex						
Male	49.9	37.6	52.4	55.1	59.6	50.9
Female	50.1	62.4	47.6	44.9	40.4	49.1
Marital status						
Married	84.4	84	82.3	80.4	75	81.3
Unmarried	10.5	10.3	12.2	14.3	19	13.3
Education						
Compulsory	44.3	43.7	44.4	45.9	47.7	45.2
Upper secondary	44.9	45.3	44.4	43.9	43	44.3
Higher	10.7	10.7	10.9	9.9	8.9	10.1
Smoking status						
Current	41.6	41.4	43.9	44.3	48.7	44
Former	33.3	32.1	31.2	33.1	33.1	32.6
Never	25.1	26.4	24.9	22.6	18.2	23.4
Recreational physical activity^1^						
Sedentary	19.1	17.9	18.4	17.4	19.8	18.5
Moderate	59.6	62.8	58.8	58.8	56	59.2
High	20.2	18.1	21.1	21.5	21.5	20.5
Vigorous	1	1.2	1.7	2.2	2.7	1.8
Occupational physical activity^2^		S				
Sedentary	22.7	22.4	23.3	22.6	22.8	22.8
Walking	43.3	48.3	42.7	41.2	38.7	42.8
Walking and lifting	20.6	19.5	20.7	21.8	23	21.1
Heavy manual	13.5	9.8	13.2	14.4	15.5	13.3
BMI	24.5 (3.6)	24.8 (3.8)	24.6 (3.5)	24.8 (3.6)	25.0 (3.6)	24.8 (3.6)
BMI ≥30	7.0	8.6	7.0	7.8	8.8	7.8
Total cholesterol (mmol/L)	6.2 (1.2)	6.2 (1.2)	6.1 (1.2)	6.1 (1.2)	6.0 (1.2)	6.1 (1.2)
Non-HDL cholesterol (mmol/L)^3^	4.7 (1.2)	4.7 (1.3)	4.7 (1.2)	4.7 (1.2)	4.6 (1.2)	4.7 (1.3)
LDL cholesterol (mmol/L)^3^	4.0 (1.1)	4.0 (1.1)	4.0 (1.1)	4.0 (1.1)	3.9 (1.1)	4.0 (1.1)
Systolic blood pressure (mm Hg)	132.5 (16.7)	131.9 (17.1)	132.9 (16.7)	133.0 (16.7)	133.2 (16.6)	132.7 (16.8)
Myocardial infarction	0.6	0.4	0.5	0.4	0.5	0.5
Stroke	0.2	0.2	0.1	0.2	0.2	0.2
Diabetes	0.8	0.6	0.8	0.7	0.6	0.7
Hypertension treatment	4.4	4.3	3.8	3.7	3.7	4.0
Energy intake (kJ/d)	7223 (2145)	6206 (2620)	7498 (2506)	7575 (2723)	7282 (2408)	7155 (2537)
Fat intake (E%)	37.7 (5.8)	37.4 (5.6)	38.2 (5.5)	37.9 (5.8)	37.4 (6.3)	37.7 (5.8)
Saturated fat intake (E%)	14.0 (3.0)	14.4 (2.8)	15.0 (3.0)	15.2 (3.3)	15.4 (3.8)	14.8 (3.2)
Trans-fatty acids intake (E%)	3.3 (1.1)	3.2 (1.1)	3.2 (1.0)	3.0 (1.0)	2.8 (1.0)	3.1 (1.0)
Cholesterol intake (mg)	237 (82)	221 (88)	263 (92)	268 (103)	267 (100)	251 (95)
Carbohydrate intake (E%)	46.9 (5.6)	46.0 (5.2)	45.4 (5.1)	45.2 (5.0)	44.8 (5.4)	45.7 (5.3)
Protein intake (E%)	14.2 (2.0)	15.4 (2.3)	15.0 (2.2)	15.6 (2.3)	16.5 (2.6)	15.3 (2.4)

Values are mean (SD) or percentage.

Abbreviation: E%, percentage of energy intake.

1 Recreational physical activity was categorized as sedentary (e.g., reading, watching TV, and other sedentary work), moderate (e.g., walking, bicycling, or other activity for a minimum of 4 h/wk), high (e.g., light sports and heavy gardening), and vigorous (e.g., hard exercise and regular competitive sports several times a week) [29].

2 Occupational physical activity was categorized as sedentary (primarily sedentary work), walking (work requiring substantial walking), walking and lifting (substantial walking and lifting), and heavy manual labor [29].

3 Available only from screening II and III.

At baseline, 95% of the participants were milk drinkers and most consumed 1–3 glasses/d. The energy-adjusted intake of milk at baseline was mean (SD) 341 (192) g [not energy adjusted = 357 (224) g]. At baseline, participants in the highest quintile of milk intake were more often males, unmarried, had lower education, were current smokers, and had slightly higher occupational physical activity, and BMI compared with those in quintile 1 (Table 1). The intake of total fat, dietary cholesterol, and protein was similar across quintiles, whereas the intake of *trans*-fatty acids and carbohydrates was highest in the lowest quintile of milk intake.

### Overall milk intake and mortality

During 2,305,642 person-years of follow-up (median: 33 years), 26,393 participants died, including 8590 from CVD, 4372 from IHD, and 3047 from AMI. With model 1, the highest compared with the lowest quintile of overall milk intake was associated with higher CVD-related, IHD-related, AMI-related, and all-cause mortality (Table 2). The associations were somewhat attenuated with additional adjustments (model 2 and 3). However, overall milk intake remained positively associated with CVD-related mortality (HR_Q5 vs Q1_: 1.12; 95% CI: 1.03, 1.21) and all-cause mortality (1.22; 95% CI 1.16, 1.28). Each 100-g/d higher overall milk intake was associated with a higher risk of CVD-related mortality (HR: 1.02; 95% CI: 1.01, 1.04) and all-cause mortality (HR: 1.04; 95% CI: 1.03, 1.05), whereas no associations were observed for IHD-related mortality (HR: 1.01; 95% CI: 0.99, 1.03) or AMI-related mortality (HR: 1.00; 95% CI: 0.98, 1.03) (Table 2).

**TABLE 2:** HRs with 95% CIs of CVD-related, IHD-related, AMI-related, and all-cause mortality per quintile and per 100 g/d of energy-adjusted cumulated mean overall milk intake (*n* = 73,860).

	Quintile of overall milk intake					
	Q1	Q2	Q3	Q4	Q5	Per 100 g/d
Median (IQR), g/d	136 (97–159)	222 (202–247)	323 (297–349)	424 (398–454)	577 (527–654)	
Person-years	430,178	475,087	468,875	466,277	465,225	2,305,642
CVD-related mortality						
Deaths (*n*)	1527	1657	1892	1795	1719	8590
Crude incidence rate (deaths/1000 person-years)	3.55	3.49	4.04	3.85	3.69	3.73
Model 1, HR (95% CI)^1^	1 (ref)	0.97 (0.91, 1.04)	1.08 (1.00, 1.15)	1.14 (1.06, 1.22)	1.24 (1.06, 1.22)	1.05 (1.01, 1.04)
Model 2, HR (95% CI)^2^	1 (ref)	0.96 (0.90, 1.04)	1.05 (0.98, 1.12)	1.06 (0.99, 1.14)	1.09 (1.01, 1.18)	1.02 (1.01, 1.03)
Model 3, HR (95% CI)^3^	1 (ref)	0.97 (0.90, 1.05)	1.06 (0.99, 1.14)	1.08 (1.00, 1.17)	1.12 (1.03, 1.21)	1.02 (1.01, 1.04)
IHD-related mortality						
Deaths (*n*)	793	803	944	924	908	4372
Crude incidence rate (deaths/1000 person-years)	1.84	1.69	2.01	1.98	1.95	1.90
Model 1, HR (95% CI)^1^	1 (ref)	0.94 (0.85, 1.04)	1.03 (0.94, 1.14)	1.11 (1.01, 1.22)	1.20 (1.09, 1.32)	1.04 (1.02, 1.06)
Model 2, HR (95% CI)^2^	1 (ref)	0.92 (0.84, 1.02)	1.00 (0.90, 1.10)	1.01 (0.91, 1.11)	1.03 (0.92, 1.14)	1.01 (0.99, 1.03)
Model 3, HR (95% CI)^3^	1 (ref)	0.93 (0.84, 1.04)	1.01 (0.91, 1.12)	1.03 (0.92, 1.14)	1.05 (0.94, 1.18)	1.01 (0.99, 1.03)
AMI-related mortality						
Deaths (*n*)	568	544	657	648	630	3047
Crude incidence rate (deaths/1000 person-years)	1.32	1.15	1.40	1.39	1.35	1.32
Model 1, HR (95% CI)^1^	1 (ref)	0.90 (0.81, 1.01)	1.00 (0.90, 1.11)	1.08 (0.97, 1.20)	1.19 (1.07, 1.33)	1.04 (1.02, 1.06)
Model 2, HR (95% CI)^2^	1 (ref)	0.87 (0.77, 0.98)	0.96 (0.86, 1.08)	0.98 (0.87, 1.11)	0.99 (0.87, 1.12)	1.00 (0.98, 1.03)
Model 3, HR (95% CI)^3^	1 (ref)	0.87 (0.77, 0.98)	0.97 (0.86, 1.09)	0.99 (0.87, 1.12)	1.00 (0.87, 1.14)	1.01 (0.98, 1.03)
All-cause mortality						
Deaths (*n*)	4631	5263	5698	5446	5355	26,393
Crude incidence rate (deaths/1000 person-years)	10.77	11.08	12.15	11.68	11.51	11.45
Model 1, HR (95% CI)^1^	1 (ref.)	1.01 (0.97, 1.05)	1.07 (1.03, 1.12)	1.13 (1.08, 1.17)	1.26 (1.21, 1.31)	1.05 (1.04, 1.05)
Model 2, HR (95% CI)^2^	1 (ref.)	1.01 (0.97, 1.05)	1.07 (1.02, 1.11)	1.09 (1.05, 1.14)	1.17 (1.12, 1.22)	1.03 (1.02, 1.04)
Model 3, HR (95% CI)^3^	1 (ref.)	1.04 (0.99, 1.08)	1.10 (1.05, 1.15)	1.13 (1.08, 1.19)	1.22 (1.16, 1.28)	1.04 (1.03, 1.05)

Abbreviations: AMI, acute myocardial infarction; CVD, cardiovascular disease; HR, hazard ratio; IHD, ischemic heart disease.

1 Model 1: Cox proportional hazards regression stratified by birth cohort (<1930, 1930–1934, 1935–1939, 1940–1944, 1945–1949, 1950–1954, and ≥1955) with age as timescale, adjusted for sex and total energy intake (kilojoules).

2 Model 2: As model 1, with additional adjustment for BMI, smoking status, recreational and occupational physical activity, education level, marital status, and comorbidities, other dietary sources of saturated fats (cheese, red and processed meat, fat on bread, meat, and fish), and intake of coffee, sweetened beverages, dietary fiber, and vitamin C (model 2).

3 Model 3: As model 2, with additional adjustment for milk types.

The HRs were similar in males and females for all outcomes (*P*-interaction = 0.28–0.99) (Supplemental Table 1). For CVD-related and IHD-related mortality, the HRs for the highest compared with the lowest intake quintile were higher in participants with BMI of 25.0–29.9 than among participants in the other BMI categories, but no interaction was found for CVD-related (*P*-interaction = 1.00), IHD-related (*P*-interaction = 0.77), and all-cause (*P*-interaction = 1.00) mortality. For AMI-related mortality, the HRs were consistently below 1 among those with BMI ≥ 30 (*P*-interaction = 0.06) (Supplemental Table 2).

### Types of milk and mortality

The characteristics of the participants at baseline stratified by types of milk consumed are shown in Supplemental Table 3. Low-fat milk consumers were more likely to be females, have higher education, and be nonsmokers compared with whole milk consumers. In contrast, whole milk consumers more frequently reported being current smokers. Of note, the proportion of individuals with BMI of ≥ 30 was highest among low-fat milk consumers (11.1%) and lowest among whole milk consumers (5.8%).

The proportions using the different types of milk at each screening are shown in Supplemental Figure 1. Use of whole milk decreased from 65.5% in screening I to 35% in screening III, in parallel with increased use of low-fat milk. When taking measurements from all available screenings into account in a mixed-effects model, consumers of whole milk had higher marginal mean concentrations of total, non-HDL, and LDL cholesterol than low-fat milk consumers (Supplemental Figure 2).

We observed a lower risk of CVD-related, IHD-related, AMI-related, and all-cause death among low-fat milk consumers than that of whole milk consumers (Table 3). In model 3, which adjusted for quantity of overall milk intake, HRs (95% CIs) for low-fat compared with whole milk consumers were 0.93 (0.88, 0.98) for CVD-related, 0.88 (0.81, 0.95) for IHD-related, 0.90 (0.82, 0.99) for AMI-related, and 0.89 (0.87, 0.92) for all-cause mortality.

**TABLE 3:** Hazard ratios (HRs) with 95% CIs of CVD-related, IHD-related, AMI-related, and all-cause mortality by use of types of milk compared with those by whole milk (*n* = 73,860).

	Type of milk			
	Whole	Mixed whole/skim	Low-fat (≤1.5% fat)	No milk
Person-years	1,008,587	265,955	920,709	110,391
CVD-related mortality				
Deaths (*n*)	3925	1064	3247	354
Model 1, HR (95% CI)^1^	1 (ref)	0.97 (0.90, 1.04)	0.93 (0.89, 0.98)	0.96 (0.86, 1.07)
Model 2, HR (95% CI)^2^	1 (ref)	1.02 (0.95, 1.10)	0.94 (0.89, 0.99)	0.97 (0.87, 1.09)
Model 3, HR (95% CI)^3^	1 (ref)	1.01 (0.94, 1.08)	0.93 (0.88, 0.98)	1.03 (0.91, 1.15)
IHD-related mortality				
Deaths (*n*)	2069	542	1584	177
Model 1, HR (95% CI)^1^	1 (ref)	0.97 (0.88, 1.07)	0.91 (0.85, 0.97)	0.98 (0.84, 1.15)
Model 2, HR (95% CI)^2^	1 (ref)	1.02 (0.93, 1.12)	0.89 (0.82, 0.96)	0.97 (0.82, 1.13)
Model 3, HR (95% CI)^3^	1 (ref)	1.01 (0.92, 1.12)	0.88 (0.81, 0.95)	0.99 (0.84, 1.17)
AMI-related mortality				
Deaths (*n*)	1452	361	1111	123
Model 1, HR (95% CI)^1^	1 (ref)	0.92 (0.82, 1.03)	0.92 (0.84, 1.00)	0.96 (0.80, 1.16)
Model 2, HR (95% CI)^2^	1 (ref)	0.97 (0.86, 1.09)	0.91 (0.83, 0.99)	0.97 (0.86, 1.09)
Model 3, HR (95% CI)^3^	1 (ref)	0.97 (0.86, 1.09)	0.90 (0.82, 0.99)	0.98 (0.80, 1.19)
All-cause mortality				
Deaths (*n*)	12,249	3141	9852	1151
Model 1, HR (95% CI)^1^	1 (ref)	0.91 (0.88, 0.95)	0.84 (0.82, 0.87)	0.93 (0.87, 0.99)
Model 2, HR (95% CI)^2^	1 (ref)	0.99 (0.95, 1.03)	0.91 (0.89, 0.94)	0.98 (0.92, 1.05)
Model 3, HR (95% CI)^3^	1 (ref)	0.97 (0.93, 1.01)	0.89 (0.87, 0.92)	1.07 (1.00, 1.14)

Abbreviations: AMI, acute myocardial infarction; CVD, cardiovascular disease; HR, hazard ratio; IHD, ischemic heart disease.

1 Model 1: Cox proportional hazards regression stratified by birth cohort (<1930, 1930–1934, 1935–1939, 1940–1944, 1945–1949, 1950–1954, and ≥1955) with age as timescale, adjusted for sex and total energy intake (kilojoules).

2 Model 2: As model 1 with additional adjustment for BMI, smoking status, recreational and occupational physical activity, education level, marital status, and comorbidities, other dietary sources of saturated fats (cheese, red and processed meat, fat on bread, meat, and fish), and intake of coffee, sweetened beverages, dietary fiber, and vitamin C.

3 Model 3: As model 2, with additional adjustment for overall milk intake amount.

**FIGURE 2:**
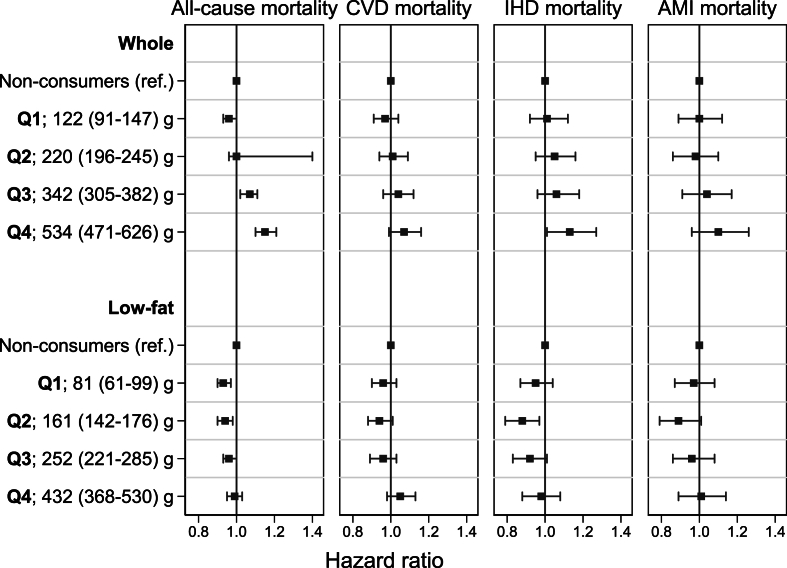
Hazard ratios and 95% CIs for all-cause, CVD-related, IHD-related, and AMI-related mortality per quartile of cumulative mean intakes of whole and low-fat milk compared with those of no intake (*n* = 73,860). Median (IQR) intake of each type of milk is shown per quartile. Nonconsumers are participants not consuming the respective type of milk or any milk. Cox proportional hazards models with age as timescale, stratified by birth cohort, and adjusted for sex and energy (kilojoules), updated BMI, smoking status, physical activity, attained education level, marital status, and comorbidities, other dietary sources of saturated fats (cheese, red and processed meat, fat on bread, meat, and fish), and intake of coffee, sweetened beverages, dietary fiber, and vitamin C. AMI, acute myocardial infarction; CVD, cardiovascular disease; IHD, ischemic heart disease.

A higher intake of whole milk was associated with increased CVD mortality; in model 2, the HR was 1.07 (95% CI: 0.99, 1.16) for quartile 4 compared with nonconsumers of whole milk (Figure 2 and Supplemental Table 4). Intake of whole milk was also positively associated with IHD-related (HR: 1.13; 95% CI: 1.01, 1.27), AMI (HR: 1.10; 95% CI: 0.96, 1.26), and all-cause mortality (HR: 1.15; 95% CI: 1.10, 1.21). Intake of low-fat milk was not associated with mortality when compared with nonconsumers of low-fat milk (Figure 2 and Supplemental Table 5). Estimates per 100 g/day intake of milk types are shown in Table 4. In the fully-adjusted model, each 100 g/day higher intake of whole milk was associated with a slightly higher risk of death of CVD-related (HR: 1.01; 95% CI: 1.00, 1.03), IHD-related (HR: 1.02; 95% CI: 1.00, 1.03), and all-cause mortality (HR: 1.03; 95% CI: 1.02, 1.04). As also shown in Table 4, each 100-g/d higher intake of low-fat milk compared with whole milk was associated with a slightly lower all-cause mortality (HR: 0.98; 95% CI: 0.97, 0.99). A similar association was found for IHD-related mortality (HR: 0.98; 95% CI: 0.96; 1.01 per 100 g/d).

**TABLE 4:** HRs with 95% CIs of CVD-related, IHD-related, AMI-related, and all-cause mortality per 100-g/d intake of whole milk and low-fat milk (*n* = 73,860).

	Whole milk	Low-fat milk	Low-fat vs whole milk
CVD-related mortality			
Model 1, HR (95% CI)^1^	1.04 (1.03, 1.05)	1.01 (1.00, 1.02)	0.98 (0.97, 1.00)
Model 2, HR (95% CI)^2^	1.01 (1.00, 1.03)	1.01 (0.99, 1.02)	1.00 (0.98, 1.01)
IHD-related mortality			
Model 1, HR (95% CI)^1^	1.04 (1.02, 1.05)	1.00 (0.98, 1.02)	0.97 (0.95, 1.00)
Model 2, HR (95% CI)^2^	1.02 (1.00, 1.03)	0.99 (0.97, 1.01)	0.98 (0.96, 1.01)
AMI-related mortality			
Model 1, HR (95% CI)^1^	1.04 (1.02, 1.05)	1.00 (0.98, 1.03)	0.98 (0.95, 1.00)
Model 2, HR (95% CI)^2^	1.01 (0.99, 1.03)	1.00 (0.97, 1.02)	0.99 (0.96, 1.02)
All-cause mortality			
Model 1, HR (95% CI)^1^	1.06 (1.05, 1.06)	0.98 (0.97, 0.99)	0.95 (0.94, 0.96)
Model 2, HR (95% CI)^2^	1.03 (1.02, 1.04)	1.00 (0.99, 1.01)	0.98 (0.97, 0.99)

Abbreviations: AMI, acute myocardial infarction; CVD, cardiovascular disease; HR, hazard ratio; IHD, ischemic heart disease.

1 Cox proportional hazards regression stratified by birth cohort (<1930, 1930–1934, 1935–1939, 1940–1944, 1945–1949, 1950–1954, and ≥1955) with age as timescale, adjusted for sex and total energy intake (kilojoules). In the substitution analysis, whole and low-fat milk were mutually adjusted for in the model.

2 Model 1, with additional adjustment for BMI, smoking status, recreational and occupational physical activity, education level, marital status, and comorbidities, other dietary sources of saturated fats (cheese, red and processed meat, fat on bread, meat, and fish), and intake of coffee, sweetened beverages, dietary fiber, and vitamin C.

Whole milk intake was more strongly associated with all mortality outcomes among females compared with those among males (*P*-interaction < 0.01 for all outcomes) (Supplemental Table 6). The positive associations between whole milk intake and all-cause mortality were also more pronounced in the subgroup with BMI < 25 (*P*-interaction = 0.01) (Supplemental Table 7). Low-fat milk intake was inversely associated with IHD-related and AMI-related mortality among females, but not those among males (*P*-interaction < 0.01 for IHD-related and *P*-interaction = 0.01 for AMI-related mortality) (Supplemental Table 8). For all-cause mortality, an interaction was observed between low-fat milk intake and BMI category, with an inverse association in those with BMI of <25 (*P*-interaction = 0.03) (Supplemental Table 9).

A strong association between whole milk intake and risk of sudden deaths from unknown causes, the secondary outcome (370 deaths) was found (HR_Q4 vs 0_: 1.80; 95% CI: 1.22, 2.66; *P*-interaction 0.001), whereas no clear associations were found with low-fat milk (Supplemental Table 10).

### Sensitivity analyses

With glasses per day as measurement unit, the direction of the associations was similar as in the main analyses using grams per day **(**Supplemental Table 11). When using only information on baseline milk intake instead of cumulative mean intakes, the estimates were attenuated for overall and whole milk, whereas the association between whole milk and IHD mortality was reversed (HR_Q4__vs 0_: 0.97; 95% CI: 0.88, 1.08) (Supplemental Table 12). The results were similar to those of the main analyses when excluding either participants with <5 y of follow-up (Supplemental Table 13), no milk consumers in the reference groups for the milk type–specific analyses (Supplemental Table 14), participants on a diet (Supplemental Table 15) or those with self-reported comorbidities (Supplemental Table 16). The main results remained unchanged when we additionally adjusted for other foods (such as bread and bread toppings) and alcoholic beverages (Supplemental Table 17).

## Discussion

The results from this large Norwegian population-based cohort, studied between 1974 and 1988, suggest a complex relationship between milk intake and mortality. Higher overall milk intake was associated with increased CVD-related and all-cause mortality. Intake of whole milk was positively associated with IHD and all-cause mortality, whereas low-fat milk intake showed no association. Notably, low-fat milk intake was associated with lower mortality when compared with whole milk intake. By using nonconsumers of specific milk types as the reference group in our quartile analyses, we aimed to assess risk associated with consuming each type of milk compared with not consuming it, which is relevant for informing public health recommendations.

The positive association with CVD and all-cause mortality for overall milk contrasts with some recent systematic reviews of cohort studies suggesting null associations [7,12], but aligns with findings from other high milk-consuming Nordic countries [35,[43], [44], [45], [46], [47]]. These discrepancies across studies may partly arise from population-specific factors, such as varying milk consumption patterns and its quantitative contribution to nutrients in the diet. For instance, a multinational cohort study with a lower average intake, found an inverse association with mortality and CVD events when comparing >1 serving/d with no milk intake [48].

The observed lower mortality associated with low-fat than that with whole milk supports a recent systematic review of cohort studies indicating positive associations between high-fat milk and all-cause and CVD-related mortality [12], and a positive association with CAD risk in another systematic review [11]. Few previous studies have directly compared whole and low-fat milk regarding mortality [13]. A Danish cohort study reported lower all-cause mortality among females, but not among males, substituting low-fat for whole milk [45]. The United States NHNES also found a lower risk of all-cause and CAD-related mortality with low-fat milk than that with whole milk [49]. Similar findings favoring low-fat milk over whole milk were observed in studies of patients with CVD and type 2 diabetes [[50], [51], [52]].

The interaction between sex and whole milk intake, with stronger associations in females, aligns with our previous studies on SFA and ruminant *trans*-fatty acids [19,40]. The stronger associations among individuals with lower BMI might reflect reverse causation or differential confounding across BMI subgroups, thus requiring cautious interpretation [53].

Milk is a valuable source of several essential nutrients but was also a major source of SFA in this cohort. SFA and *trans*-fatty acids raise serum LDL cholesterol and apolipoprotein B, which are key atherogenic risk factors [54,55]. Intake of SFA was also positively associated with CVD-related and all-cause mortality in this cohort [40]. These associations were stronger for palmitic and myristic acid, the predominant SFAs in dairy fat [40]. Milk is also a source of ruminant *trans*-fatty acids, which were associated with CVD-related and CAD-related mortality among females in this cohort [19]. The milk fat content may thus be a plausible explanation of the positive associations with mortality, particularly IHD-related mortality. However, a Swedish cohort study found differential associations of fat from milk and fat from cheese with all-cause mortality, suggesting an interaction between dairy fat and other components [44]. Isocaloric substitutions of dairy fat with vegetable fat, PUFA, or carbohydrates from whole grains have been associated with lower risk of CVD and CVD-related mortality in United States cohort studies [56,57]. Thus, the displacement of unsaturated fats or whole grain foods for whole milk might play a role in our findings.

Strengths of this study include its large sample size and high attendance rate (>80 %), long follow-up, and large number of cases. The participants were considered broadly representative of the eligible population during the study period, and the relatively young age at baseline likely limited reverse causation [20]. Repeated dietary assessments captured changes in milk intake during a period of substantial dietary transitions, thereby limiting measurement error and regression dilution bias [33,58]. Linkage with the Cause of Death Registry ensured practically complete ascertainment of mortality outcomes.

However, several potential limitations warrant discussion. First, diet was self-reported, potentially introducing misreporting. Comparison with a 24HR indicated that the FFQ could reliably differentiate between users of different milk types, but discrepancies for ≤25% of the participants could introduce uncertainty into our findings [28]. However, a single 24HR is an imperfect standard due to day-to-day variability and potential misreporting. Furthermore, the consistency of associations between reported milk types and serum lipids as expected from fat content lends some support of the validity of the exposure data. Nonetheless, the possibility of overestimation or underestimation of the quantity of milk consumed cannot be excluded. Because the FFQ was not distributed in all municipalities in screening I, many participants lacked data from all 3 diet assessments, which may have led to an underestimation of the associations [58]. A further limitation is that we did not have updated milk consumption data after screening III.

Second, milk as an ingredient in composite foods was not included in the quantification of milk intake, likely resulting in an underestimation of total milk intake. Accordingly, our findings primarily concern the intake of milk as a beverage. We lacked information on the proportions of milk consumed as fresh (nonfermented) or soured (fermented), which might have different health effects [35,46,47], although fermented milk consumption was relatively low in Norway during the study period [59,60].

Third, residual and unmeasured confounding cannot be excluded despite having detailed information on key covariates. Comorbidities were self-reported, and we had no data on medication use during follow-up. The increased use of lipid-lowering medications from ∼2000 onward might have modified risk of CVD-related mortality with time.

Finally, the study was limited to 3 counties in Norway during 1974–1988, which might restrict the generalizability of findings to other populations and contemporary settings due to secular changes in dietary patterns and risk profiles. The historical context of the study, conducted during a period of declining milk fat intake and serum cholesterol concentrations in Norway, is therefore a central aspect [61].

In conclusion, associations between milk intake and CVD-related and all-cause mortality varied by type of milk, with positive associations found for whole milk and a modestly inverse association with IHD-related and all-cause mortality was found for low-fat milk when compared with whole milk. Repeated dietary assessments accounting for changes in milk intake were important to avoid misclassification of different types of milk during the 1970s and 1980s. Further mechanistic studies comparing the effects of different types of milk on CVD risk factors are needed for a more robust causal interpretation.

## Author contributions

The authors’ responsibilities were as follows – EKA: designed the study with contributions from JJC and MHC. EKA, KR: obtained ethics approval and source data; IL: calculated nutrient intakes; EKA: conducted data analyses and wrote the first draft of the article; IL, MBV: provided statistical advice; KR: is the guarantor; and all authors: contributed to the interpretation of the results and critical revision of the manuscript and approved the final version. The corresponding author attests that all listed authors meet authorship criteria and that no others meeting the criteria have been omitted.

## Data availability

The data included in these analyses cannot be openly shared by the authors because they were subject to ethical approval. Access to data can be applied for through the Norwegian Institute of Public Health, https://www.fhi.no/en/more/access-to-data/.

## Funding

The Norwegian Counties Study was funded by the National Health Screening Service, Oslo, Norway. No financial support or sponsorship was received for the conduct of this study or the preparation of this manuscript.

## Conflict of interest

JJC reports personal fees from Novo Nordisk, outside of the submitted work. KR reports personal fees from Amgen, Amarin, Bayer, Norwegian Directorate of Health, Norwegian Medicines Agency, Norwegian Medical Association, and Sanofi, outside the submitted work. The other authors report no support from any organization for the submitted work; no financial relationships with any organizations that might have an interest in the submitted work in the previous 3 y; no other relationships or activities that could appear to have influenced the submitted work.
