# Supplementary material for: Low-fat and whole milk consumption in relation to cardiovascular disease–related and all-cause mortality: a prospective cohort study in 3 Norwegian counties
Source: Am J Clin Nutr. 2025 Aug 7;122(4):1075–85. doi: 10.1016/j.ajcnut.2025.07.035 (PMC12674078; doi:10.1016/j.ajcnut.2025.07.035)
Supplement: Supplementary file 1 [file mmc1.docx]

**Low-fat and Whole Milk Consumption in relation to Cardiovascular and All-Cause Mortality: The Norwegian Counties Study**

Erik Kristoffer Arnesen et al.

**Supplementary material**

[**Supplementary Figure 1.** Proportions using the different milk types at each screening.	3](#_Toc198476393)

[**Supplementary Figure 2.** Estimated marginal means of total, non-HDL and LDL cholesterol from mixed-effects models, adjusted for age, sex, BMI, smoking status, physical activity, education, other dietary sources of SFA, coffee, and sweetened beverages, as well as cumulative average intakes of energy intake, total amount of milk, dietary fiber and vitamin C (mg/1000 kcal). Total cholesterol, n = 73,800, non-HDL cholesterol, n = 62,629, and LDL-cholesterol, n = 60,457; non-HDL and LDL cholesterol was only available for screenings II and III.	4](#_Toc198476394)

[**Supplementary Table 1.** Hazard ratios (HRs) with 95% confidence intervals (CIs) of cardiovascular disease (CVD), ischemic heart disease (IHD), acute myocardial infarction (AMI) and all-cause mortality per quintile of energy-adjusted cumulated mean overall milk intake, stratified by sex (n = 73,860).	5](#_Toc198476395)

[**Supplementary Table 2.** Hazard ratios (HRs) with 95% confidence intervals (CIs) of cardiovascular disease (CVD), ischemic heart disease (IHD), acute myocardial infarction (AMI), and all-cause mortality per quintile of energy-adjusted cumulated mean overall milk intake, stratified by BMI category (n = 73,860).	6](#_Toc198476396)

[**Supplementary Table 3.** Baseline characteristics according to type of milk consumed at baseline in the Norwegian Counties Study (n = 73,860).	7](#_Toc198476397)

[**Supplementary Table 4.** Hazard ratios (HRs) with 95% confidence intervals (CIs) of cardiovascular disease (CVD), ischemic heart disease (IHD), acute myocardial infarction (AMI), and all-cause mortality per quartile of energy-adjusted cumulated mean intakes of whole milk (n = 73,860).	9](#_Toc198476398)

[**Supplementary Table 5.** Hazard ratios (HRs) with 95% confidence intervals (CIs) of cardiovascular disease (CVD), ischemic heart disease (IHD), acute myocardial infarction (AMI), and all-cause mortality per quartile of energy-adjusted cumulated mean intakes of low-fat (≤1.5% fat) milk (n = 73,860).	10](#_Toc198476399)

[**Supplementary Table 6.** Hazard ratios (HRs) with 95% confidence intervals (CIs) of cardiovascular disease (CVD), ischemic heart disease (IHD), acute myocardial infarction (AMI), and all-cause mortality per quartile of whole milk intake, stratified by sex (n = 73,860).	11](#_Toc198476400)

[**Supplementary Table 7.** Hazard ratios (HRs) with 95% confidence intervals (CIs) of cardiovascular disease (CVD), ischemic heart disease (IHD), acute myocardial infarction (AMI), and all-cause mortality per quartile of whole milk intake, stratified by BMI category (n = 73,860).	12](#_Toc198476401)

[**Supplementary Table 8.** Hazard ratios (HRs) with 95% confidence intervals (CIs) of cardiovascular disease (CVD), ischemic heart disease (IHD), acute myocardial infarction (AMI), and all-cause mortality per quartile of low-fat (≤1.5% fat) milk intake, stratified by sex (n = 73,860).	13](#_Toc198476402)

[**Supplementary Table 9.** Hazard ratios (HRs) with 95% confidence intervals (CIs) of cardiovascular disease (CVD), ischemic heart disease (IHD), acute myocardial infarction (AMI), and all-cause mortality per quartile of low-fat (≤1.5% fat) milk intake, stratified by BMI category (n = 73,860).	14](#_Toc198476403)

[**Supplementary Table 10.** Hazard ratios (HRs) with 95% confidence intervals (CIs) of sudden deaths from unknown causes by cumulative mean intake of overall, whole, and low-fat milk (n = 73,860).	15](#_Toc198476404)

[**Supplementary Table 11.** Hazard ratios (HRs) with 95% confidence intervals (CIs) of all-cause, cardiovascular disease (CVD), ischemic heart disease (IHD), and acute myocardial infarction (AMI) mortality by intake of milk in glasses/day (n = 73,860).	16](#_Toc198476405)

[**Supplementary Table 12.** Hazard ratios (HRs) with 95% confidence intervals (CIs) of all-cause, cardiovascular disease (CVD), ischemic heart disease (IHD), and acute myocardial infarction (AMI) mortality by only baseline milk intake instead of cumulative mean intakes from all screenings (n = 73,860).	17](#_Toc198476406)

[**Supplementary Table 13.** Hazard ratios (HRs) with 95% confidence intervals (CIs) of all-cause, cardiovascular disease (CVD), ischemic heart disease (IHD), and acute myocardial infarction (AMI) mortality by milk intake, excluding the first 5 years of follow-up.	19](#_Toc198476407)

[**Supplementary Table 14.** Hazard ratios (HRs) with 95% confidence intervals (CIs) of cardiovascular disease (CVD), ischemic heart disease (IHD), acute myocardial infarction (AMI), and all-cause mortality by milk intake, excluding non-milk consumers in the reference group in milk type analyses (n = 71,132).	21](#_Toc198476408)

[**Supplementary Table 15.** Hazard ratios (HRs) with 95% confidence intervals (CIs) of cardiovascular disease (CVD), ischemic heart disease (IHD), acute myocardial infarction (AMI), and all-cause mortality by milk intake, excluding participants reporting to be on a diet (N = 72,282).	23](#_Toc198476409)

[**Supplementary Table 16.** Hazard ratios (HRs) with 95% confidence intervals (CIs) of cardiovascular disease (CVD), ischemic heart disease (IHD), acute myocardial infarction (AMI), and all-cause mortality by milk intake, excluding participants with self-reported comorbidities (N = 70,025).	25](#_Toc198476410)

[**Supplementary Table 17.** Hazard ratios (HRs) with 95% confidence intervals (CIs) of cardiovascular disease (CVD), ischemic heart disease (IHD), acute myocardial infarction (AMI), and all-cause mortality by milk intake, additionally adjusted for intake of bread, jam, syrup/honey, mayonnaise-based salads, chocolate spreads, and fish as bread toppings (e.g., herring, sardines), eggs, and beer/wine/liquor (n = 72,977).	27](#_Toc198476411)

## **Supplementary Figure 1.** Proportions using the different milk types at each screening.

## **Supplementary Figure 2.** Estimated marginal means of total, non-HDL and LDL cholesterol from mixed-effects models, adjusted for age, sex, BMI, smoking status, physical activity, education, other dietary sources of SFA, coffee, and sweetened beverages, as well as cumulative average intakes of energy intake, total amount of milk, dietary fiber and vitamin C (mg/1000 kcal). Total cholesterol, n = 73,800, non-HDL cholesterol, n = 62,629, and LDL-cholesterol, n = 60,457; non-HDL and LDL cholesterol was only available for screenings II and III. HDL, high-density lipoprotein; LDL, low-density lipoprotein.

## **Supplementary Table 1.** Hazard ratios (HRs) with 95% confidence intervals (CIs) of cardiovascular disease (CVD), ischemic heart disease (IHD), acute myocardial infarction (AMI) and all-cause mortality per quintile of energy-adjusted cumulated mean overall milk intake, stratified by sex (n = 73,860).

|  | Quintile of overall milk intake^1^ | | | | | *p*_interaction_^2^ |
| --- | --- | --- | --- | --- | --- | --- |
|  | Q1 | Q2 | Q3 | Q4 | Q5 |  |
| Median (IQR), g/day | 136 (97–159) | 222 (202–247) | 323 (297–349) | 424 (398–454) | 577 (527–654) |  |
| CVD mortality | | | | | | 0.98 |
| Males | 1 (ref) | 0.97 (0.88, 1.07) | 1.06 (0.97, 1.17) | 1.08 (0.98, 1.18) | 1.08 (0.98, 1.19) |  |
| Females | 1 (ref) | 1.01 (0.89, 1.14) | 1.06 (0.93, 1.20) | 1.08 (0.95, 1.24) | 1.16 (1.00, 1.34) |  |
| IHD mortality | | | | | | 0.99 |
| Males | 1 (ref) | 0.95 (0.84, 1.08) | 1.04 (0.92, 1.17) | 1.04 (0.92, 1.18) | 1.06 (0.93, 1.21) |  |
| Females | 1 (ref) | 0.91 (0.75, 1.11) | 0.94 (0.77, 1.15) | 1.00 (0.81, 1.23) | 0.99 (0.79, 1.25) |  |
| AMI mortality | | | | | | 0.83 |
| Males | 1 (ref) | 0.85 (0.73, 0.99) | 0.99 (0.86, 1.14) | 0.98 (0.84, 1.13) | 0.98 (0.84, 1.14) |  |
| Females | 1 (ref) | 0.92 (0.73, 1.16) | 0.93 (0.73, 1.18) | 1.04 (0.81, 1.33) | 1.05 (0.80, 1.38) |  |
| All-cause mortality |  |  |  |  |  | 0.28 |
| Males | 1 (ref) | 1.04 (0.98, 1.10) | 1.09 (1.03, 1.15) | 1.10 (1.04, 1.16) | 1.19 (1.12, 1.26) |  |
| Females | 1 (ref) | 1.07 (1.00, 1.14) | 1.12 (1.04, 1.20) | 1.19 (1.10, 1.28) | 1.23 (1.14, 1.34) |  |

^1^ Cox proportional hazards regression stratified by birth cohort (<1930, 1930-34, 1935-39, 1940-44, 1945-49, 1950-54, and ≥1955) with age as timescale, adjusted for total energy intake (kJ), BMI (kg/m^2^), smoking status, recreational and occupational physical activity, education level, marital status, and comorbidities, type of milk, other dietary sources of saturated fats (cheese, red and processed meat, fat on bread, meat, and fish), intake of coffee, sweetened beverages, dietary fiber, and vitamin C. AMI, acute myocardial infarction; CVD, cardiovascular disease; HR, hazard ratio; IHD, ischemic heart disease; IQR, interquartile range.

^2^ From likelihood ratio test comparing the model with cross-product term for sex and milk to the model without.

## **Supplementary Table 2.** Hazard ratios (HRs) with 95% confidence intervals (CIs) of cardiovascular disease (CVD), ischemic heart disease (IHD), acute myocardial infarction (AMI), and all-cause mortality per quintile of energy-adjusted cumulated mean overall milk intake, stratified by BMI category (n = 73,860).

|  | Quintile of overall milk intake^1^ | | | | | *p*_interaction_^2^ |
| --- | --- | --- | --- | --- | --- | --- |
|  | Q1 | Q2 | Q3 | Q4 | Q5 |  |
| Median (IQR), g/day | 136 (97–159) | 222 (202–247) | 323 (297–349) | 424 (398–454) | 577 (527–654) |  |
| CVD mortality | | | | |  | 1.00 |
| <25.0 kg/m^2^ | 1 (ref) | 1.00 (0.89, 1.11) | 1.08 (0.97, 1.21) | 1.06 (0.94, 1.19) | 1.09 (0.95, 1.23) |  |
| 25.0–29.9 kg/m^2^ | 1 (ref) | 1.03 (0.92, 1.16) | 1.10 (0.98, 1.23) | 1.16 (1.03, 1.31) | 1.21 (1.07, 1.36) |  |
| ≥30 kg/m^2^ | 1 (ref) | 0.81 (0.66, 0.98) | 0.92 (0.75, 1.11) | 0.90 (0.73, 1.10) | 0.91 (0.74, 1.12) |  |
| IHD mortality | | | | | | 0.77 |
| <25.0 kg/m^2^ | 1 (ref) | 0.96 (0.82, 1.13) | 1.02 (0.86, 1.19) | 1.06 (0.90, 1.26) | 1.06 (0.90, 1.26) |  |
| 25.0–29.9 kg/m^2^ | 1 (ref) | 1.04 (0.88, 1.22) | 1.07 (0.91, 1.25) | 1.09 (0.93, 1.28) | 1.17 (0.99 1.38) |  |
| ≥30 kg/m^2^ | 1 (ref) | 0.67 (0.51, 0,88) | 0.87 (0.67, 1.13) | 0.79 (0.60, 1.04) | 0.74 (0.56, 0.99) |  |
| AMI mortality | | | | |  | 0.06 |
| <25.0 kg/m^2^ | 1 (ref) | 0.98 (0.80, 1.20) | 1.03 (0.85, 1.26) | 1.15 (0.94, 1.41) | 1.08 (0.87, 1.35) |  |
| 25.0–29.9 kg/m^2^ | 1 (ref) | 0.93 (0.77, 1.12) | 0.98 (0.82, 1.18) | 0.98 (0.81, 1.19) | 1.08 (0.88, 1.31) |  |
| ≥30 kg/m^2^ | 1 (ref) | 0.54 (0.39, 0.74) | 0.78 (0.58, 1.06) | 0.69 (0.50, 0.95) | 0.64 (0.46, 0.89) |  |
| All-cause mortality | |  |  |  |  | 1.00 |
| <25.0 kg/m^2^ | 1 (ref) | 1.05 (0.98, 1.11) | 1.09 (1.02, 1.16) | 1.11 (1.04, 1.19) | 1.23 (1.14, 1.31) |  |
| 25.0–29.9 kg/m^2^ | 1 (ref) | 1.06 (0.99, 1.13) | 1.13 (1.05, 1.21) | 1.18 (1.10, 1.27) | 1.23 (1.15, 1.33) |  |
| ≥30 kg/m^2^ | 1 (ref) | 0.96 (0.84, 1.09) | 1.05 (0.92, 1.19) | 1.03 (0.90, 1.17) | 1.06 (0.93, 1.22) |  |

^1^ Cox proportional hazards regression stratified by birth cohort (<1930, 1930-34, 1935-39, 1940-44, 1945-49, 1950-54, and ≥1955) with age as timescale, adjusted for sex, total energy intake (kJ), smoking status, recreational and occupational physical activity, education level, marital status, and comorbidities, type of milk, other dietary sources of saturated fats (cheese, red and processed meat, fat on bread, meat, and fish), intake of coffee, sweetened beverages, dietary fiber, and vitamin C. AMI, acute myocardial infarction; BMI, body mass index; CVD, cardiovascular disease; HR, hazard ratio. IHD, ischemic heart disease; IQR, interquartile range.

^2^ From likelihood ratio test comparing the model with cross-product term for sex and milk to the model without.

## **Supplementary Table 3.** Baseline characteristics according to type of milk consumed at baseline in the Norwegian Counties Study (n = 73,860).

| Type of milk | Whole (3.8% fat) | Mixed whole/skimmed | Low-fat  (≤1.5% fat) | No milk | Total |
| --- | --- | --- | --- | --- | --- |
| *N* | 40,876 | 10,048 | 19,319 | 3,617 | 73,860 |
| Overall milk intake (grams/day (energy-adjusted), mean (SD) | 338 (167) | 377 (182) | 393 (197) | 0 (0) | 341 (192) |
| Age (years), mean (SD) | 40.7 (7.4) | 41.6 (7.4) | 41.9 (7.3) | 41.5 (7.1) | 41.2 (7.4) |
| Sex, %  Male  Female | 57.2  42.8 | 48.2  51.8 | 42.0  58.0 | 34.9  65.1 | 50.9  49.1 |
| Marital status, %  Married | 81.4 | 80.3 | 81.1 | 83.5 | 81.3 |
| Education, %  Compulsory  Upper secondary/vocational training  University | 49.7  42.8  7.3 | 44.2  44.3  11.2 | 36.2  47.5  15.7 | 44.8  44.5  10.6 | 45.2  44.3  10.1 |
| Smoking status, % |  |  |  |  |  |
| Current  Former  Never | 50.1  26.2  23.6 | 38.4  30.6  30.9 | 19.4  47.0  33.6 | 44.7  32.8  22.5 | 44.0  32.6  23.4 |
| Recreational physical activity^1^, % |  |  |  |  |  |
| Sedentary  Moderate  High  Vigorous | 19.7  57.6  20.9  1.8 | 15.6  60.6  22.1  1.7 | 16.8  61.7  19.5  2.0 | 22.4  60.1  16.8  0.7 | 18.5  59.2  20.5  1.8 |
| Occupational physical activity^2^, % |  |  |  |  |  |
| Sedentary  Walking  Walking and lifting  Heavy manual | 20.3  40.6  22.7  16.3 | 21.0  45.5  20.1  13.5 | 28.8  45.2  18.6  7.4 | 23.2  47.5  19.5  9.8 | 22.8  42.8  21.1  13.3 |
| BMI (kg/m^2^), mean (SD) | 24.3 (3.4) | 25.2 (3.7) | 25.5 (3.7) | 24.6 (3.9) | 24.8 (3.6) |
| BMI ≥30 kg/m^2^, % | 5.8 | 9.5 | 11.1 | 9.1 | 7.9 |
| Total cholesterol (mmol/l), mean (SD) | 6.1 (1.2) | 6.1 (1.2) | 6.1 (1.2) | 6.1 (1.3) | 6.1 (1.2) |
| Non-HDL cholesterol (mmol/l), mean (SD)^3^ | 4.8 (1.3) | 4.7 (1.2) | 4.6 (1.2) | 4.7 (1.3) | 4.7 (1.3) |
| LDL cholesterol (mmol/l), mean (SD)^3^ | 4.0 (1.1) | 4.0 (1.1) | 3.9 (1.1) | 4.0 (1.1) | 4.0 (1.1) |
| Systolic blood pressure (mmHg), mean (SD) | 132.9 (16.4) | 133.0 (17.1) | 132.3 (17.2) | 131.9 (17.2) | 132.7 (16.8) |
| Myocardial infarction, % |  |  |  |  |  |
| Stroke, % | 0.1 | 0.2 | 0.3 | 0.2 | 0.2 |
| Diabetes, % | 0.6 | 0.7 | 1.0 | 0.7 | 0.7 |
| Hypertension treatment, % | 2.8 | 4.5 | 5.9 | 5.8 | 4.0 |
| Energy intake, mean (SD), kJ/day | 7817 (2565) | 7001 (2313) | 6098 (2119) | 5755 (2290) | 7155 (2537) |
| Fat intake, mean (SD), E% | 40.0 (5.0) | 36.4 (4.8) | 33.8 (5.4) | 36.6 (6.3) | 37.7 (5.8) |
| Saturated fat intake, mean (SD), E% | 16.3 (2.9) | 13.8 (2.3) | 12.3 (2.1) | 13.3 (3.0) | 14.8 (3.2) |
| Trans fat intake, mean (SD), E% | 3.3 (1.0) | 3.1 (1.0) | 2.8 (1.0) | 3.2 (1.2) | 3.1 (1.0) |
| Cholesterol intake, mean (SD), mg | 283 (98) | 236 (79) | 202 (68) | 197 (81) | 251 (95) |
| Carbohydrate intake, mean (SD), E% | 44.3 (5.0) | 46.5 (4.7) | 47.7 (5.1) | 47.3 (6.1) | 45.7 (5.3) |
| Protein intake, mean (SD), E% | 14.5 (1.8) | 15.8 (2.1) | 17.1 (2.6) | 14.7 (2.5) | 15.3 (2.4) |

^1^ Recreational physical activity categorized as sedentary (reading, watching TV, other sedentary work), moderate (walking, bicycling, or other activity for min. 4 hours/week), high (light sports, heavy gardening, etc.), and vigorous (hard exercise, regular competitive sports several times a week)(27).
^2^ Occupational physical activity categorized as sedentary (primary sedentary work), walking (work requiring substantial walking), walking and lifting (substantial walking and lifting), and heavy manual labor (27).
^3^ Available only in screening II and III.
BMI, Body mass index; HDL, high-density lipoprotein; LDL, low-density lipoprotein; kJ, kiloJoules; E%, percentage of energy intake

## **Supplementary Table 4.** Hazard ratios (HRs) with 95% confidence intervals (CIs) of cardiovascular disease (CVD), ischemic heart disease (IHD), acute myocardial infarction (AMI), and all-cause mortality per quartile of energy-adjusted cumulated mean intakes of whole milk (n = 73,860).

|  |  | Quartile of milk intake^1^ | | | |
| --- | --- | --- | --- | --- | --- |
|  | Non-consumers | Q1 | Q2 | Q3 | Q4 |
| Median (IQR), g/day | 0 | 122 (91–147) | 220 (196–245) | 342 (305–382) | 534 (471–626) |
| CVD mortality | | | | | |
| Person-years | 591,509 | 482,899 | 435,890 | 414,049 | 381,296 |
| Deaths, n | 1641 | 2033 | 1737 | 1734 | 1445 |
| Model 1, HR (95% CI)^1^ | 1 (ref.) | 0.99 (0.93, 1.06) | 1.06 (0.99, 1.13) | 1.13 (1.05, 1.21) | 1.22 (1.13, 1.32) |
| Model 2, HR (95% CI)^2^ | 1 (ref.) | 0.97 (0.91, 1.04) | 1.01 (0.94, 1.09) | 1.04 (0.96, 1.12) | 1.07 (0.99, 1.16) |
| IHD mortality | | | | | |
| Deaths, n | 793 | 1013 | 877 | 891 | 798 |
| Model 1, HR (95% CI)^1^ | 1 (ref.) | 1.01 (0.92, 1.11) | 1.08 (0.98, 1.19) | 1.13 (1.03, 1.25) | 1.27 (1.14, 1.40) |
| Model 2, HR (95% CI)^2^ | 1 (ref.) | 1.01 (0.92, 1.12) | 1.05 (0.95, 1.16) | 1.06 (0.96, 1.18) | 1.13 (1.01, 1.27) |
| AMI mortality | | | | | |
| Deaths, n | 565 | 707 | 587 | 625 | 563 |
| Model 1, HR (95% CI)^1^ | 1 (ref.) | 0.99 (0.89, 1.11) | 1.02 (0.90, 1.14) | 1.12 (0.99, 1.26) | 1.25 (1.11, 1.42) |
| Model 2, HR (95% CI)^2^ | 1 (ref.) | 1.00 (0.89, 1.12) | 0.98 (0.86, 1.10) | 1.04 (0.91, 1.17) | 1.10 (0.96, 1.26) |
| All-cause mortality |  |  |  |  |  |
| Deaths, n | 5250 | 6010 | 5176 | 5300 | 4657 |
| Model 1, HR (95% CI)^1^ | 1 (ref.) | 1.00 (0.96, 1.03) | 1.07 (1.03, 1.11) | 1.19 (1.14, 1.24) | 1.35 (1.30, 1.41) |
| Model 2, HR (95% CI)^2^ | 1 (ref.) | 0.96 (0.93, 1.00) | 1.00 (0.96, 1.04) | 1.07 (1.02, 1.11) | 1.15 (1.10, 1.21) |

^1^ Cox proportional hazards regression stratified by birth cohort (<1930, 1930-34, 1935-39, 1940-44, 1945-49, 1950-54, and ≥1955) with age as timescale, adjusted for sex and total energy intake (kJ). “Non-consumers” are participants not consuming the respective type of milk or any milk. AMI, acute myocardial infarction; CVD, cardiovascular disease; HR, hazard ratio; IHD, ischemic heart disease; IQR, interquartile range.
^2^ Model 1 with additional adjustment for BMI (kg/m^2^), smoking status, recreational and occupational physical activity, education level, marital status, and comorbidities, other dietary sources of saturated fats (cheese, red and processed meat, fat on bread, meat, and fish), intake of coffee, sweetened beverages, dietary fiber, and vitamin C.

## **Supplementary Table 5.** Hazard ratios (HRs) with 95% confidence intervals (CIs) of cardiovascular disease (CVD), ischemic heart disease (IHD), acute myocardial infarction (AMI), and all-cause mortality per quartile of energy-adjusted cumulated mean intakes of low-fat (≤1.5% fat) milk (n = 73,860).

|  |  | Quartile of milk intake^1^ | | | |
| --- | --- | --- | --- | --- | --- |
|  | Non-consumers | Q1 | Q2 | Q3 | Q4 |
| Median (IQR), g/day | 0 | 81 (61–99) | 161 (142–176) | 252 (221–285) | 432 (368–530) |
| CVD mortality | | | | | |
| Person-years | 1,030,186 | 323,311 | 308,075 | 316,130 | 327,972 |
| Deaths, n | 3789 | 1435 | 1132 | 1191 | 1043 |
| Model 1, HR (95% CI)^1^ | 1 (ref.) | 0.92 (0.87, 0.98) | 0.91 (0.85, 0.97) | 0.95 (0.89, 1.01) | 1.05 (0.98, 1.12) |
| Model 2, HR (95% CI)^2^ | 1 (ref.) | 0.96 (0.90, 1.03) | 0.94 (0.88, 1.01) | 0.96 (0.89, 1.03) | 1.05 (0.98, 1.13) |
| IHD mortality | | | | | |
| Deaths, n | 2007 | 711 | 539 | 594 | 521 |
| Model 1, HR (95% CI)^1^ | 1 (ref.) | 0.92 (0.84, 1.00) | 0.86 (0.78, 0.95) | 0.94 (0.85, 1.03) | 1.01 (0.91, 1.11) |
| Model 2, HR (95% CI)^2^ | 1 (ref.) | 0.95 (0.87, 1.04) | 0.88 (0.79-0.97) | 0.92 (0.83, 1.01) | 0.98 (0.88, 1.08) |
| AMI mortality | | | | | |
| Deaths, n | 1392 | 494 | 374 | 421 | 366 |
| Model 1, HR (95% CI)^1^ | 1 (ref.) | 0.93 (0.83, 1.03) | 0.87 (0.77, 0.98) | 0.97 (0.86, 1.08) | 1.03 (0.92, 1.16) |
| Model 2, HR (95% CI)^2^ | 1 (ref.) | 0.97 (0.87, 1.08) | 0.89 (0.79, 1.01) | 0.96 (0.86, 1.08) | 1.01 (0.89, 1.14) |
| All-cause mortality |  |  |  |  |  |
| Deaths, n | 11,927 | 4264 | 3466 | 3645 | 3091 |
| Model 1, HR (95% CI)^1^ | 1 (ref.) | 0.86 (0.83, 0.89) | 0.85 (0.82, 0.89) | 0.89 (0.86, 0.93) | 0.91 (0.87, 0.95) |
| Model 2, HR (95% CI)^2^ | 1 (ref.) | 0.93 (0.90, 0.97) | 0.94 (0.90, 0.98) | 0.96 (0.93, 1.00) | 0.99 (0.95, 1.03) |

^1^ Cox proportional hazards regression stratified by birth cohort (<1930, 1930-34, 1935-39, 1940-44, 1945-49, 1950-54, and ≥1955) with age as timescale, adjusted for sex and total energy intake (kJ). “Non-consumers” are participants not consuming the respective type of milk or any milk. AMI, acute myocardial infarction; CVD, cardiovascular disease; HR, hazard ratio; IHD, ischemic heart disease; IQR, interquartile range.
^2^ Model 1 with additional adjustment for BMI (kg/m^2^), smoking status, recreational and occupational physical activity, education level, marital status, and comorbidities, other dietary sources of saturated fats (cheese, red and processed meat, fat on bread, meat, and fish), intake of coffee, sweetened beverages, dietary fiber, and vitamin C.

## **Supplementary Table 6.** Hazard ratios (HRs) with 95% confidence intervals (CIs) of cardiovascular disease (CVD), ischemic heart disease (IHD), acute myocardial infarction (AMI), and all-cause mortality per quartile of whole milk intake, stratified by sex (n = 73,860).

| Sex | Quartile of milk intake^1^ | | | | |  |
| --- | --- | --- | --- | --- | --- | --- |
|  | Non-consumers | Q1 | Q2 | Q3 | Q4 | *p*_interaction_^2^ |
| Median (IQR), g/day | 0 | 122 (91–147) | 220 (196–245) | 342 (305–382) | 534 (471–626) |  |
| CVD mortality | | | | | | <0.01 |
| Males | 1 (Ref) | 0.96 (0.88, 1.05) | 0.98 (0.90, 1.08) | 1.02 (0.93, 1.12) | 1.04 (0.94, 1.15) |  |
| Females | 1 (Ref) | 1.00 (0.90, 1.10) | 1.04 (0.93, 1.16) | 1.03 (0.91, 1.17) | 1.12 (0.97, 1.30) |  |
| IHD mortality | | | | | | <0.01 |
| Males | 1 (Ref) | 0.96 (0.86, 1.09) | 1.00 (0.88, 1.13) | 1.02 (0.90, 1.15) | 1.05 (0.92, 1.19) |  |
| Females | 1 (Ref) | 1.11 (0.94, 1.32) | 1.12 (0.94, 1.35) | 1.10 (0.90, 1.35) | 1.42 (1.13, 1.79) |  |
| AMI mortality | | | | | | <0.01 |
| Males | 1 (ref) | 0.97 (0.84, 1.12) | 0.94 (0.81, 1.09) | 1.02 (0.88, 1.18) | 1.02 (0.87, 1.19) |  |
| Females | 1 (ref) | 1.02 (0.84, 1.24) | 1.02 (0.83, 1.26) | 1.01 (0.80, 1.28) | 1.41 1.08, 1.83) |  |
| All-cause mortality | | | | | | <0.01 |
| Males | 1 (Ref) | 0.97 (0.92, 1.02) | 0.97 (0.92, 1.03) | 1.04 (0.98, 1.10) | 1.11 (1.05, 1.18) |  |
| Females | 1 (Ref) | 0.96 (0.91, 1.01) | 1.01 (0.95, 1.07) | 1.08 (1.02, 1.16) | 1.19 (1.11, 1.29) |  |

^1^ Cox proportional hazards regression stratified by birth cohort (<1930, 1930-34, 1935-39, 1940-44, 1945-49, 1950-54, and ≥1955) with age as timescale, adjusted for total energy intake (kJ), BMI (kg/m^2^), smoking status, recreational and occupational physical activity, education level, marital status, and comorbidities, other dietary sources of saturated fats (cheese, red and processed meat, fat on bread, meat, and fish), intake of coffee, sweetened beverages, dietary fiber, and vitamin C. “Non-consumers” are participants not consuming the respective type of milk or any milk. AMI, acute myocardial infarction; CVD, cardiovascular disease; HR, hazard ratio; IHD, ischemic heart disease; IQR, interquartile range.

^2^ From likelihood ratio test comparing the model with cross-product term for sex and milk to the model without.

## **Supplementary Table 7.** Hazard ratios (HRs) with 95% confidence intervals (CIs) of cardiovascular disease (CVD), ischemic heart disease (IHD), acute myocardial infarction (AMI), and all-cause mortality per quartile of whole milk intake, stratified by BMI category (n = 73,860).

| BMI category | Quartile of milk intake^1^ | | | | |  |
| --- | --- | --- | --- | --- | --- | --- |
|  | Non-consumers | Q1 | Q2 | Q3 | Q4 | *p*_interaction_^2^ |
| Median (IQR), g/day | 0 | 122 (91–147) | 220 (196–245) | 342 (305–382) | 534 (471–626) |  |
| CVD mortality | | | |  |  | 0.34 |
| <25.0 | 1 (ref) | 1.04 (0.92, 1.16) | 1.07 (0.95, 1.20) | 1.14 (1.01, 1.29) | 1.14 (1.00, 1.30) |  |
| 25.0–29.9 | 1 (ref) | 0.93 (0.84, 1.03) | 0.96 (0.86, 1.06) | 0.98 (0.87, 1.09) | 1.01 (0.89, 1.14) |  |
| ≥30 | 1 (ref) | 1.03 (0.89, 1.21) | 1.09 (0.91, 1.29) | 1.00 (0.83, 1.20) | 1.05 (0.85, 1.30) |  |
| IHD mortality |  |  |  |  |  | 0.31 |
| <25.0 | 1 (ref) | 1.13 (0.95, 1.34) | 1.17 (0.98, 1.39) | 1.13 (0.94, 1.35) | 1.26 (1.04, 1.52) |  |
| 25.0–29.9 | 1 (ref) | 0.95 (0.82, 1.09) | 0.93 (0.80, 1.08) | 1.03 (0.89, 1.21) | 1.00 (0.85, 1.19) |  |
| ≥30 | 1 (ref) | 1.05 (0.85, 1.31) | 1.16 (0.91, 1.47) | 1.02 (0.79, 1.32) | 1.18 (0.88, 1.57) |  |
| AMI mortality | | | | | | 0.75 |
| <25.0 | 1 (ref) | 1.12 (0.91, 1.38) | 1.09 (0.88, 1.34) | 1.12 (0.91, 1.40) | 1.28 (1.02, 1.61) |  |
| 25.0–29.9 | 1 (ref) | 0.97 (0.82, 1.14) | 0.90 (0.76, 1.08) | 1.02 (0.85, 1.22) | 0.98 (0.80, 1.20) |  |
| ≥30 | 1 (ref) | 0.92 (0.71, 1.20) | 1.03 (0.78, 1.37) | 0.97 (0.71, 1.31) | 1.08 (0.77, 1.52) |  |
| All-cause mortality | | | | | | 0.01 |
| <25.0 | 1 (ref) | 1.02 (0.96, 1.08) | 1.04 (0.98, 1.11) | 1.11 (1.05, 1.19) | 1.23 (1.15, 1.31) |  |
| 25.0–29.9 | 1 (ref) | 0.93 (0.88, 0.99) | 0.96 (0.90, 1.02) | 1.05 (0.98, 1.12) | 1.09 (1.01, 1.17) |  |
| ≥30 | 1 (ref) | 0.98 (0.89, 1.08) | 1.04 (0.94, 1.16) | 1.01 (0.90, 1.14) | 1.08 (0.95, 1.23) |  |

^1^ Cox proportional hazards regression stratified by birth cohort (<1930, 1930-34, 1935-39, 1940-44, 1945-49, 1950-54, and ≥1955) with age as timescale, adjusted for sex, total energy intake (kJ), smoking status, recreational and occupational physical activity, education level, marital status, and comorbidities, other dietary sources of saturated fats (cheese, red and processed meat, fat on bread, meat, and fish), intake of coffee, sweetened beverages, dietary fiber, and vitamin C. “Non-consumers” are participants not consuming the respective type of milk or any milk. AMI, acute myocardial infarction; BMI, body mass index; CVD, cardiovascular disease; HR, hazard ratio; IHD, ischemic heart disease; IQR, interquartile range.
^2^ From likelihood ratio test comparing the model with cross-product term for sex and milk to the model without.

## **Supplementary Table 8.** Hazard ratios (HRs) with 95% confidence intervals (CIs) of cardiovascular disease (CVD), ischemic heart disease (IHD), acute myocardial infarction (AMI), and all-cause mortality per quartile of low-fat (≤1.5% fat) milk intake, stratified by sex (n = 73,860).

| Sex |  | Quartile of milk intake^1^ | | |  |  |
| --- | --- | --- | --- | --- | --- | --- |
|  | Non-consumers | Q1 | Q2 | Q3 | Q4 | *p*_interaction_^2^ |
| Median (IQR), g/day | 0 | 81 (61–99) | 161 (142–176) | 252 (221–285) | 432 (368–530) |  |
| CVD mortality | | | | | | <0.01 |
| Males | 1 (ref) | 0.99 (0.91, 1.07) | 0.94 (0.86, 1.02) | 0.99 (0.95, 1.14) | 1.04 (0.95, 1.14) |  |
| Females | 1 (ref) | 0.91 (0.82, 1.01) | 0.93 (0.84, 1.04) | 0.88 (0.78, 0.99) | 1.01 (0.89, 1.15) |  |
| IHD mortality | | | | | | <0.01 |
| Males | 1 (ref) | 0.94 (0.84, 1.05) | 0.93 (0.83, 1.05) | 0.98 (0.87, 1.10) | 1.01 (0.89, 1.14) |  |
| Females | 1 (ref) | 0.93 (0.80, 1.10) | 0.77 (0.64, 0.92) | 0.77 (0.64, 0.92) | 0.83 (0.68, 1.03) |  |
| AMI mortality | | | | | | 0.01 |
| Males | 1 (ref) | 0.95 (0.83, 1.08) | 0.96 (0.83, 1.11) | 1.02 (0.89, 1.17) | 1.04 (0.90, 1.20) |  |
| Females | 1 (ref) | 0.96 (0.80, 1.16) | 0.76 (0.61, 0.94) | 0.82 (0.66, 1.02) | 0.89 (0.70, 1.14) |  |
| All-cause mortality | | | | | | 0.20 |
| Males | 1 (ref) | 0.95 (0.90, 1.00) | 0.95 (0.90, 1.01) | 0.97 (0.92, 1.02) | 0.99 (0.93, 1.04) |  |
| Females | 1 (ref) | 0.91 (0.86, 0.96) | 0.92 (0.87, 0.98) | 0.94 (0.89, 1.00) | 0.97 (0.91, 1.04) |  |

^1^ Cox proportional hazards regression stratified by birth cohort (<1930, 1930-34, 1935-39, 1940-44, 1945-49, 1950-54, and ≥1955) with age as timescale, adjusted for total energy intake (kJ), BMI (kg/m^2^), smoking status, recreational and occupational physical activity, education level, marital status, and comorbidities, other dietary sources of saturated fats (cheese, red and processed meat, fat on bread, meat, and fish), intake of coffee, sweetened beverages, dietary fiber, and vitamin C. “Non-consumers” are participants not consuming the respective type of milk or any milk. AMI, acute myocardial infarction; CVD, cardiovascular disease; HR, hazard ratio; IHD, ischemic heart disease; IQR, interquartile range.

^2^ From likelihood ratio test comparing the model with cross-product term for sex and milk to the model without.

## **Supplementary Table 9.** Hazard ratios (HRs) with 95% confidence intervals (CIs) of cardiovascular disease (CVD), ischemic heart disease (IHD), acute myocardial infarction (AMI), and all-cause mortality per quartile of low-fat (≤1.5% fat) milk intake, stratified by BMI category (n = 73,860).

| BMI category |  | Quartile of milk intake^1^ | | | |  |
| --- | --- | --- | --- | --- | --- | --- |
|  | Non-consumers | Q1 | Q2 | Q3 | Q4 | *p*_interaction_^2^ |
| Median (IQR), g/day | 0 | 81 (61–99) | 161 (142–176) | 252 (221–285) | 432 (368–530) |  |
| CVD mortality | | |  |  |  | 0.70 |
| 25.0 | 1 (ref) | 0.98 (0.89, 1.09) | 0.91 (0.81, 1.02) | 0.94 (0.84, 1.06) | 1.00 (0.87, 1.14) |  |
| 25.0, 29.9 | 1 (ref) | 0.98 (0.89, 1.08) | 0.98 (0.89, 1.09) | 0.99 (0.90, 1.10) | 1.10 (0.99, 1.23) |  |
| ≥30 | 1 (ref) | 0.90 (0.76, 1.07) | 0.92 (0.77, 1.09) | 0.93 (0.79, 1.11) | 1.02 (0.86, 1.21) |  |
| IHD mortality | | | |  |  | 0.24 |
| <25.0 | 1 (ref) | 0.98 (0.85, 1.13) | 0.92 (0.78, 1.09) | 0.96 (0.81, 1.14) | 0.96 (0.79, 1.17) |  |
| 25.0, 29.9 | 1 (ref) | 1.00 (0.88, 1.14) | 0.87 (0.75, 1.101) | 0.93 (0.80, 1.06) | 1.08 (0.93, 1.25) |  |
| ≥30 | 1 (ref) | 0.79 (0.62, 0.99) | 0.82 (0.64, 1.04) | 0.84 (0.67, 1.06) | 0.77 (0.61, 0.98) |  |
| AMI mortality | |  |  |  |  | 0.27 |
| <25.0 | 1 (ref) | 0.99 (0.83, 1.17) | 0.90 (0.74, 1.11) | 1.01 (0.82, 1.23) | 0.96 (0.75, 1.21) |  |
| 25.0–29.9 | 1 (ref) | 1.04 (0.89, 1.21) | 0.94 (0.79, 1.12) | 0.97 (0.82, 1.15) | 1.16 (0.97, 1.38) |  |
| ≥30 | 1 (ref) | 0.75 (0.57, 1.00) | 0.75 (0.56, 1.00) | 0.85 (0.65, 1.11) | 0.76 (0.57, 1.00) |  |
| All-cause mortality | | | | | | 0.03 |
| <25.0 | 1 (ref) | 0.93 (0.88, 0.98) | 0.92 (0.87, 0.98) | 0.94 (0.88, 1.00) | 0.93 (0.87, 1.00) |  |
| 25.0–29.9 | 1 (ref) | 0.96 (0.90, 1.01) | 0.97 (0.92, 1.04) | 1.00 (0.94, 1.06) | 1.03 (0.96, 1.09) |  |
| ≥30 | 1 (ref) | 0.90 (0.81, 1.00) | 0.91 (0.82, 1.02) | 0.95 (0.86, 1.06) | 0.99 (0.89, 1.10) |  |

^1^ Cox proportional hazards regression stratified by birth cohort (<1930, 1930-34, 1935-39, 1940-44, 1945-49, 1950-54, and ≥1955) with age as timescale, adjusted for sex, total energy intake (kJ), smoking status, recreational and occupational physical activity, education level, marital status, and comorbidities, other dietary sources of saturated fats (cheese, red and processed meat, fat on bread, meat, and fish), intake of coffee, sweetened beverages, dietary fiber, and vitamin C. “Non-consumers” are participants not consuming the respective type of milk or any milk. AMI, acute myocardial infarction; BMI, body mass index; CVD, cardiovascular disease; HR, hazard ratio; IHD, ischemic heart disease; IQR, interquartile range.

^2^ From likelihood ratio test comparing the model with cross-product term for sex and milk to the model without.

## **Supplementary Table 10.** Hazard ratios (HRs) with 95% confidence intervals (CIs) of sudden deaths from unknown causes by cumulative mean intake of overall, whole, and low-fat milk (n = 73,860).

|  | Median (IQR), g/day | Deaths, n | HR (95% CI)^1^ |
| --- | --- | --- | --- |
| Overall milk |  |  |  |
| Q1 | 136 (97–159) | 63 | 1 (ref.) |
| Q2 | 222 (202–247) | 78 | 1.14 (0.80, 1.63) |
| Q3 | 323 (297–349) | 61 | 0.83 (0.57, 1.22) |
| Q4 | 424 (398–454) | 86 | 1.22 (0.85, 1.76) |
| Q5 | 577 (527–654) | 82 | 1.23 (0.83, 1.81) |
| Per 100 g |  |  | 1.08 (1.00, 1.15) |
| Whole milk |  |  |  |
| Non-consumers | 0 | 54 | 1 (ref.) |
| Q1 | 122 (91–147) | 71 | 1.12 (0.78, 1.63) |
| Q2 | 220 (196–245) | 76 | 1.36 (0.94, 1.96) |
| Q3 | 342 (305–382) | 78 | 1.36 (0.93, 1.98) |
| Q4 | 534 (471–626) | 91 | 1.80 (1.22, 2.66) |
| Per 100 g |  |  | 1.10 (1.04, 1.17) |
| Low-fat milk |  |  |  |
| Non-consumers | 0 | 199 | 1 (ref.) |
| Q1 | 81 (61–99) | 57 | 0.93 (0.68, 1.27) |
| Q2 | 161 (142–176) | 42 | 0.86 (0.61, 1.22) |
| Q3 | 252 (221–285) | 36 | 0.70 (0.48, 1.02) |
| Q4 | 432 (368–530) | 36 | 0.80 (0.55, 1.18) |
| Per 100 g |  |  | 0.94 (0.87, 1.02) |

^1^ Cox proportional hazards regression stratified by birth cohort (<1930, 1930-34, 1935-39, 1940-44, 1945-49, 1950-54, and ≥1955) with age as timescale, adjusted for sex, total energy intake (kJ), BMI (kg/m2), smoking status, recreational and occupational physical activity, education level, marital status, and comorbidities, other dietary sources of saturated fats (cheese, red and processed meat, fat on bread, meat, and fish), intake of coffee, sweetened beverages, dietary fiber, and vitamin C. HRs for overall milk are also adjusted for type of milk. “Non-consumers” are participants not consuming the respective type of milk or any milk. HR, hazard ratio; IQR, interquartile range.

## **Supplementary Table 11.** Hazard ratios (HRs) with 95% confidence intervals (CIs) of all-cause, cardiovascular disease (CVD), ischemic heart disease (IHD), and acute myocardial infarction (AMI) mortality by intake of milk in glasses/day (n = 73,860).

|  | CVD mortality | | IHD mortality | AMI mortality | All-cause mortality |
| --- | --- | --- | --- | --- | --- |
| Milk type | HR (95% CI)^1^ | | HR (95% CI)^1^ | HR (95% CI)^1^ | HR (95% CI)^1^ |
| Overall milk | |  |  |  |  |
| <1 glass | 1 (ref.) | | 1 (ref.) | 1 (ref.) | 1 (ref.) |
| 1 glass | 0.92 (0.82, 1.03) | | 0.88 (0.75, 1.04) | 0.93 (0.77, 1.13) | 0.88 (0.82, 0.93) |
| 2 glasses | 0.99 (0.88, 1.11) | | 0.93 (0.79, 1.10) | 0.95 (0.78, 1.15) | 0.95 (0.89, 1.02) |
| 3 glasses | 1.02 (0.90, 1.15) | | 0.96 (0.81, 1.14) | 0.95 (0.78, 1.17) | 0.99 (0.93, 1.06) |
| ≥4 glasses | 1.03 (0.91, 1.17) | | 0.94 (0.79, 1.12) | 0.97 (0.79, 1.20) | 1.03 (0.96, 1.11) |
| Per glass/day | 1.03 (1.01, 1.05) | | 1.02 (0.99, 1.05) | 1.02 (0.98, 1.05) | 1.04 (1.03, 1.05) |
| Whole milk | |  |  |  |  |
| <1 glass | 1 (ref.) | | 1 (ref.) | 1 (ref.) | 1 (ref.) |
| 1–<2 glasses | 1.06 (1.00, 1.12) | | 1.13 (1.04, 1.23) | 1.06 (0.96, 1.18) | 1.05 (1.02, 1.09) |
| 2–<3 glasses | 1.13 (1.06, 1.21) | | 1.16 (1.05, 1.27) | 1.17 (1.04, 1.31) | 1.13 (1.08, 1.17) |
| 3–<4 glasses | 1.10 (1.01, 1.20) | | 1.12 (0.99, 1.25) | 1.08 (0.93, 1.24) | 1.15 (1.09, 1.20) |
| ≥4 glasses | 1.10 (1.01, 1.20) | | 1.13 (1.01, 1.26) | 1.11 (0.97, 1.27) | 1.19 (1.14, 1.25) |
| Per glass/day | 1.03 (1.01, 1.04) | | 1.03 (1.01, 1.05) | 1.03 (1.00, 1.05) | 1.04 (1.03, 1.05) |
| Low-fat milk | |  |  |  |  |
| <1 glass | 1 (ref.) | | 1 (ref.) | 1 (ref.) | 1 (ref.) |
| 1–<2 glasses | 0.97 (0.91, 1.03) | | 0.93 (0.85, 1.01) | 0.91 (0.82, 1.01) | 0.94 (0.90, 0.97) |
| 2–<3 glasses | 0.98 (0.91, 1.04) | | 0.94 (0.86, 1.03) | 0.93 (0.84, 1.05) | 0.95 (0.92, 0.99) |
| 3–<4 glasses | 0.96 (0.89, 1.05) | | 0.92 (0.81, 1.03) | 0.91 (0.79, 1.05) | 0.94 (0.90, 0.99) |
| ≥4 glasses | 1.02 (0.94, 1.11) | | 0.93 (0.83, 1.05) | 0.96 (0.84, 1.11) | 0.98 (0.93, 1.03) |
| Per glass/day | 1.00 (0.99, 1.02) | | 0.98 (0.96, 1.01) | 0.99 (0.96, 1.02) | 0.99 (0.98, 1.00) |

^1^ Cox proportional hazards regression stratified by birth cohort (<1930, 1930-34, 1935-39, 1940-44, 1945-49, 1950-54, and ≥1955) with age as timescale, adjusted for sex, total energy intake (kJ), BMI (kg/m^2^), smoking status, recreational and occupational physical activity, education level, marital status, and comorbidities, other dietary sources of saturated fats (cheese, red and processed meat, fat on bread, meat, and fish), intake of coffee, sweetened beverages, dietary fiber, and vitamin C. HRs for overall milk are also adjusted for type of milk. “Non-consumers” are participants not consuming the respective type of milk or any milk. AMI, acute myocardial infarction; CVD, cardiovascular disease; HR, hazard ratio; IHD, ischemic heart disease.

## **Supplementary Table 12.** Hazard ratios (HRs) with 95% confidence intervals (CIs) of all-cause, cardiovascular disease (CVD), ischemic heart disease (IHD), and acute myocardial infarction (AMI) mortality by only baseline milk intake instead of cumulative mean intakes from all screenings (n = 73,860).

|  | Median (IQR), g/day | CVD mortality | IHD mortality | AMI mortality | All-cause mortality |
| --- | --- | --- | --- | --- | --- |
| Milk type | | HR (95% CI)^1^ | HR (95% CI)^1^ | HR (95% CI)^1^ | HR (95% CI)^1^ |
| Overall milk | | |  |  |  |
| Q1 | 132 (56–153) | 1 (ref.) | 1 (ref.) | 1 (ref.) | 1 (ref.) |
| Q2 | 215 (197–238) | 0.98 (0.92, 1.06) | 1.03 (0.93, 1.13) | 0.95 (0.85, 1.07) | 1.01 (0.97, 1.05) |
| Q3 | 323 (293–352) | 1.02 (0.95, 1.09) | 1.03 (0.93, 1.13) | 1.00 (0.89, 1.12) | 1.06 (1.02, 1.10) |
| Q4 | 428 (401–459) | 0.99 (0.92, 1.06) | 1.00 (0.90, 1.10) | 0.98 (0.87, 1.10) | 1.06 (1.01, 1.10) |
| Q5 | 594 (540–678) | 1.07 (1.00, 1.16) | 1.07 (0.96, 1.18) | 1.03 (0.91, 1.16) | 1.17 (1.12, 1.22) |
| Per 100 g/d | | 1.01 (1.00, 1.03) | 1.01 (0.99, 1.02) | 1.00 (0.98, 1.02) | 1.03 (1.02, 1.04) |
| Whole milk, median (IQR) g/day | | |  |  |  |
| Non-consumers | | 1 (ref.) | 1 (ref.) | 1 (ref.) | 1 (ref.) |
| Q1 | 136 (64–166) | 0.97 (0.91, 1.04) | 1.01 (0.92, 1.11) | 1.00 (0.89, 1.12) | 0.97 (0.93, 1.01) |
| Q2 | 236 (208–266) | 0.98 (0.92, 1.05) | 1.04 (0.94, 1.14) | 0.97 (0.87, 1.09) | 1.00 (0.97, 1.04) |
| Q3 | 375 (331–414) | 0.99 (0.92, 1.06) | 1.02 (0.92, 1.12) | 0.99 (0.88, 1.12) | 1.02 (0.98, 1.06) |
| Q4 | 573 (506–673) | 0.95 (0.88, 1.02) | 0.97 (0.88, 1.08) | 0.92 (0.81, 1.04) | 1.07 (1.03, 1.12) |
| Per 100 g/d | | 0.99 (0.98, 1.00) | 0.99 (0.98, 1.01) | 0.99 (0.97, 1.00) | 1.01 (1.00, 1.02) |
| Low-fat milk, median (IQR) g/day | | |  |  |  |
| Non-consumers | | 1 (ref.) | 1 (ref.) | 1 (ref.) | 1 (ref.) |
| Q1 | 130 (88–148) | 1.02 (0.95, 1.09) | 0.98 (0.88, 1.09) | 1.00 (0.89, 1.13) | 0.97 (0.93, 1.01) |
| Q2 | 197 (180–228) | 1.00 (0.93, 1.08) | 0.96 (0.86, 1.07) | 1.01 (0.89, 1.15) | 0.99 (0.95, 1.04) |
| Q3 | 328 (293–357) | 1.11 (1.02, 1.20) | 1.10 (0.98, 1.23) | 1.14 (1.00, 1.30) | 1.03 (0.98, 1.08) |
| Q4 | 514 (452–615) | 1.09 (1.00, 1.18) | 0.99 (0.88, 1.12) | 1.03 (0.89, 1.19) | 1.04 (0.99, 1.09) |
| Per 100 g/d | | 1.02 (1.00, 1.03) | 1.00 (0.99, 1.02) | 1.01 (0.99, 1.03) | 1.01 (1.00, 1.01) |

^1^ Cox proportional hazards regression stratified by birth cohort (<1930, 1930-34, 1935-39, 1940-44, 1945-49, 1950-54, and ≥1955) with age as timescale, adjusted for sex, total energy intake (kJ), BMI (kg/m^2^), smoking status, recreational and occupational physical activity, education level, marital status, and comorbidities, other dietary sources of saturated fats (cheese, red and processed meat, fat on bread, meat, and fish), intake of coffee, sweetened beverages, dietary fiber, and vitamin C. HRs for overall milk are also adjusted for type of milk. “Non-consumers” are participants not consuming the respective type of milk or any milk.
AMI, acute myocardial infarction; CVD, cardiovascular disease; HR, hazard ratio; IHD, ischemic heart disease; IQR, interquartile range.

## **Supplementary Table 13.** Hazard ratios (HRs) with 95% confidence intervals (CIs) of all-cause, cardiovascular disease (CVD), ischemic heart disease (IHD), and acute myocardial infarction (AMI) mortality by milk intake, excluding the first 5 years of follow-up.

|  | Median (IQR), g/day | CVD mortality | | | IHD mortality | | AMI mortality | | All-cause mortality | |
| --- | --- | --- | --- | --- | --- | --- | --- | --- | --- | --- |
| Milk type | | Person-years | Deaths, N | HR (95% CI)^1^ | Deaths, N | HR (95% CI)^1^ | Deaths, N | HR (95% CI)^1^ | Deaths, N | HR (95% CI)^1^ |
| Overall milk | | | | | | | | | | |
| Q1 | 136 (97–159) | 373,835 | 1503 | 1 (ref.) | 776 | 1 (ref.) | 555 | 1 (ref.) | 4553 | 1 (ref.) |
| Q2 | 222 (202–247) | 422,945 | 1636 | 0.98 (0.90, 1.05) | 792 | 0.94 (0.85, 1.05) | 534 | 0.87 (0.77, 0.99) | 5196 | 1.04 (1.00, 1.09) |
| Q3 | 323 (297–349) | 418,860 | 1858 | 1.06 (0.98, 1.14) | 925 | 1.01 (0.91, 1.12) | 640 | 0.96 (0.85, 1.09) | 5604 | 1.10 (1.05, 1.15) |
| Q4 | 424 (398–454) | 411,787 | 1762 | 1.08 (1.00, 1.17) | 904 | 1.03 (0.93, 1.15) | 629 | 0.99 (0.87, 1.12) | 5341 | 1.13 (1.08, 1.18) |
| Q5 | 577 (527–654) | 404,406 | 1676 | 1.11 (1.03, 1.21) | 880 | 1.06 (0.94, 1.18) | 607 | 1.00 (0.87, 1.14) | 5217 | 1.21 (1.16, 1.27) |
| Per 100 g/d | |  | | 1.02 (1.00, 1.04) |  | 1.01 (0.99, 1.03) |  | 1.01 (0.98, 1.03) |  | 1.04 (1.03, 1.05) |
| Whole milk | | | | | | | | | | |
| Non-consumers | 0 | 511,583 | 1604 | 1 (ref.) | 771 | 1 (ref.) | 545 | 1 (ref.) | 5122 | 1 (ref.) |
| Q1 | 122 (91–147) | 447,494 | 2010 | 0.97 (0.90, 1.04) | 998 | 1.00 (0.91, 1.10) | 695 | 0.99 (0.88, 1.11) | 5957 | 0.96 (0.93, 1.00) |
| Q2 | 220 (196–245) | 388,751 | 1715 | 1.01 (0.94, 1.08) | 862 | 1.04 (0.94, 1.15) | 573 | 0.97 (0.86, 1.10) | 5107 | 1.00 (0.96, 1.04) |
| Q3 | 342 (305–382) | 363,991 | 1702 | 1.03 (0.96, 1.11) | 873 | 1.06 (0.95, 1.17) | 611 | 1.04 (0.91, 1.18) | 5208 | 1.07 (1.02, 1.11) |
| Q4 | 534 (471–626) | 320,014 | 1404 | 1.06 (0.98, 1.15) | 773 | 1.13 (1.00, 1.27) | 541 | 1.10 (0.96, 1.26) | 4517 | 1.15 (1.10, 1.20) |
| Per 100 g/d | |  | | 1.01 (1.00, 1.03) |  | 1.02 (1.00, 1.04) |  | 1.00 (0.99, 1.03) |  | 1.03 (1.02, 1.04) |
| Low-fat milk | | | | | | | | | | |
| Non-consumers | 0 | 863,744 | 3700 | 1 (ref.) | 1956 | 1 (ref.) | 1348 | 1 (ref.) | 11,636 | 1 (ref.) |
| Q1 | 81 (61–99) | 310,190 | 1426 | 0.96 (0.90, 1.02) | 704 | 0.94 (0.86, 1.03) | 488 | 0.95 (0.86, 1.06) | 4238 | 0.93 (0.89, 0.96) |
| Q2 | 161 (142–176) | 208,350 | 1118 | 0.94 (0.87, 1.01) | 529 | 0.87 (0.78, 0.96) | 366 | 0.88 (0.78, 1.00) | 3427 | 0.94 (0.90, 0.97) |
| Q3 | 252 (221–285) | 289,283 | 1174 | 0.95 (0.89, 1.02) | 584 | 0.91 (0.82, 1.00) | 414 | 0.95 (0.85, 1.07) | 3594 | 0.96 (0.92, 1.00) |
| Q4 | 432 (368–530) | 288,266 | 1017 | 1.05 (0.97, 1.13) | 504 | 0.97 (0.87, 1.08) | 349 | 0.99 (0.87, 1.13) | 3016 | 0.99 (0.95, 1.03) |
| Per 100 g/d | |  | | 1.01 (0.99, 1.02) |  | 0.99 (0.97, 1.01) |  | 0.99 (0.97, 1.02) |  | 1.00 (0.99, 1.01) |

^1^ Cox proportional hazards regression stratified by birth cohort (<1930, 1930-34, 1935-39, 1940-44, 1945-49, 1950-54, and ≥1955) with age as timescale, adjusted for sex, total energy intake (kJ), BMI (kg/m^2^), smoking status, recreational and occupational physical activity, education level, marital status, and comorbidities, other dietary sources of saturated fats (cheese, red and processed meat, fat on bread, meat, and fish), intake of coffee, sweetened beverages, dietary fiber, and vitamin C. HRs for overall milk are also adjusted for type of milk. “Non-consumers” are participants not consuming the respective type of milk or any milk.
AMI, acute myocardial infarction; CVD, cardiovascular disease; HR, hazard ratio; IHD, ischemic heart disease; IQR, interquartile range.

## **Supplementary Table 14.** Hazard ratios (HRs) with 95% confidence intervals (CIs) of cardiovascular disease (CVD), ischemic heart disease (IHD), acute myocardial infarction (AMI), and all-cause mortality by milk intake, excluding non-milk consumers in the reference group in milk type analyses (n = 71,132).

|  | Median (IQR), g/day | CVD mortality | | | IHD mortality | | | | | AMI mortality | | | | | All-cause mortality | | |  |  |
| --- | --- | --- | --- | --- | --- | --- | --- | --- | --- | --- | --- | --- | --- | --- | --- | --- | --- | --- | --- |
| Milk type |  | Person-years | Deaths, N | HR (95% CI)^1^ | Deaths, N | HR (95% CI)^1^ | | | | Deaths, N | HR (95% CI)^1^ | | | | Deaths, N | HR (95% CI)^1^ | |  |  |
| Overall milk | | |  | | |  |  |  |  | | |  |  |  | | |  | | |
| Q1 | 147 (127–164) | 429,964 | 1503 | 1 (ref.) | 776 | 1 (ref.) | | | | 555 | 1 (ref.) | | | | 4556 | 1 (ref.) | |  |  |
| Q2 | 222 (202–247) | 474,901 | 1636 | 0.98 (0.91, 1.05) | 792 | 0.93 (0.84, 1.03) | | | | 534 | 0.85 (0.75, 0.97) | | | | 5198 | 1.04 (0.99, 1.08) | |  |  |
| Q3 | 323 (297–349) | 468,616 | 1858 | 1.07 (0.99, 1.15) | 925 | 1.01 (0.91, 1.12) | | | | 640 | 0.96 (0.85, 1.09) | | | | 5604 | 1.10 (1.05, 1.15) | |  |  |
| Q4 | 424 (398–454) | 466,008 | 1765 | 1.10 (1.01, 1.18) | 904 | 1.04 (0.94, 1.16) | | | | 629 | 0.99 (0.87, 1.13) | | | | 5343 | 1.14 (1.09, 1.19) | |  |  |
| Q5 | 577 (527–654) | 464,833 | 1677 | 1.11 (1.03, 1.21) | 881 | 1.04 (0.93, 1.17) | | | | 608 | 0.98 (0.86, 1.12) | | | | 5221 | 1.21 (1.16, 1.27) | |  |  |
| Per 100 g/d | |  | | 1.03 (1.01, 1.04) |  | 1.01 (0.99, 1.03) | | | |  | 1.00 (0.98, 1.03) | | | |  | 1.04 (1.03, 1.05) | |  |  |
| Whole milk | | |  |  |  |  | | | |  |  | | | |  |  | |  |  |
| Non-consumers | 0 | 591,153 | 1604 | 1 (ref.) | 771 | 1 (ref.) | | | | 545 | 1 (ref.) | | | | 5123 | 1 (ref.) | |  |  |
| Q1 | 123 (93–148) | 482,728 | 2010 | 0.98 (0.91, 1.05) | 998 | 1.02 (0.92, 1.12) | | | | 695 | 1.00 (0.89, 1.13) | | | | 5959 | 0.97 (0.94, 1.01) | |  |  |
| Q2 | 220 (196–245) | 435,633 | 1716 | 1.02 (0.95, 1.10) | 862 | 1.05 (0.95, 1.17) | | | | 573 | 0.98 (0.87, 1.11) | | | | 5110 | 1.01 (0.97, 1.05) | |  |  |
| Q3 | 342 (305–382) | 413,958 | 1703 | 1.04 (0.97, 1.13) | 874 | 1.07 (0.96, 1.19) | | | | 612 | 1.05 (0.93, 1.20) | | | | 5210 | 1.08 (1.03, 1.12) | |  |  |
| Q4 | 534 (471–626) | 380,851 | 1404 | 1.07 (0.99, 1.16) | 773 | 1.14 (1.02, 1.28) | | | | 541 | 1.11 (0.97, 1.28) | | | | 4520 | 1.16 (1.10, 1.21) | |  |  |
| Per 100 g/d | |  | | 1.01 (1.00, 1.03) |  | 1.02 (1.00, 1.04) | | | |  | 1.01 (0.99, 1.03) | | | |  | 1.03 (1.02, 1.04) | |  |  |
| Low-fat milk | | |  |  |  |  | | | |  |  | | | |  |  | |  |  |
| Non-consumers | 0 | 1,029,382 | 3700 | 1 (ref.) | 1956 | 1 (ref.) | | | | 1348 | 1 (ref.) | | | | 11,645 | 1 (ref.) | |  |  |
| Q1 | 81 (62–99) | 323,245 | 1426 | 0.96 (0.90, 1.03) | 704 | 0.95 (0.87, 1.03) | | | | 488 | 0.96 (0.86, 1.07) | | | | 4238 | 0.93 (0.90, 0.97) | |  |  |
| Q2 | 161 (142–176) | 307,980 | 1118 | 0.94 (0.88, 1.01) | 529 | 0.87 (0.79, 0.96) | | | | 366 | 0.88 (0.78, 1.00) | | | | 3427 | 0.94 (0.90, 0.98) | |  |  |
| Q3 | 252 (221–285) | 315,988 | 1175 | 0.96 (0.89, 1.02) | 584 | 0.91 (0.83, 1.00) | | | | 414 | 0.96 (0.85, 1.08) | | | | 3595 | 0.96 (0.92, 1.00) | |  |  |
| Q4 | 432 (368–530) | 327,728 | 1018 | 1.04 (0.97, 1.13) | 505 | 0.96 (0.87, 1.07) | | | | 350 | 0.99 (0.87, 1.12) | | | | 3017 | 0.98 (0.94, 1.02) | |  |  |
| Per 100 g/d | |  | | 1.01 (0.99, 1.02) |  | 0.99 (0.97, 1.01) | | | |  | 0.99 (0.97, 1.02) | | | |  | 1.00 (0.99, 1.01) | |  |  |

^1^Cox proportional hazards regression stratified by birth cohort (<1930, 1930-34, 1935-39, 1940-44, 1945-49, 1950-54, and ≥1955) with age as timescale, adjusted for sex, total energy intake (kJ), BMI (kg/m2), smoking status, recreational and occupational physical activity, education level, marital status, and comorbidities, other dietary sources of saturated fats (cheese, red and processed meat, fat on bread, meat, and fish), intake of coffee, sweetened beverages, dietary fiber, and vitamin C. HRs for overall milk are also adjusted for type of milk. “Non-consumers” are participants not consuming the respective type of milk or any milk.
AMI, acute myocardial infarction; CVD, cardiovascular disease; HR, hazard ratio; IHD, ischemic heart disease; IQR, interquartile range.

## **Supplementary Table 15.** Hazard ratios (HRs) with 95% confidence intervals (CIs) of cardiovascular disease (CVD), ischemic heart disease (IHD), acute myocardial infarction (AMI), and all-cause mortality by milk intake, excluding participants reporting to be on a diet (N = 72,282).

|  | Median (IQR), g/day | CVD mortality | | | IHD mortality | | AMI mortality | | All-cause mortality | |
| --- | --- | --- | --- | --- | --- | --- | --- | --- | --- | --- |
| Milk type |  | Person-years | Deaths, N | HR (95% CI)^1^ | Deaths, N | HR (95% CI)^1^ | Deaths, N | HR (95% CI)^1^ | Deaths, N | HR (95% CI)^1^ |
| Overall milk | |  |  |  |  |  |  |  |  |  |
| Q1 | 136 (97–159) | 419,924 | 1457 | 1 (ref.) | 754 | 1 (ref.) | 539 | 1 (ref.) | 4461 | 1 (ref.) |
| Q2 | 222 (202–247) | 462,681 | 1587 | 0.98 (0.91, 1.06) | 766 | 0.94 (0.84, 1.05) | 522 | 0.89 (0.78, 1.01) | 5038 | 1.04 (0.99, 1.08) |
| Q3 | 323 (297–349) | 457,233 | 1807 | 1.08 (1.00, 1.16) | 892 | 1.01 (0.91, 1.13) | 622 | 0.98 (0.87, 1.11) | 5455 | 1.10 (1.05, 1.15) |
| Q4 | 424 (398–454) | 455,024 | 1717 | 1.09 (1.01, 1.18) | 883 | 1.03 (0.93, 1.15) | 620 | 1.01 (0.89, 1.15) | 5248 | 1.14 (1.09, 1.19) |
| Q5 | 576 (527–653) | 452,295 | 1644 | 1.14 (1.05, 1.23) | 862 | 1.06 (0.95, 1.19) | 604 | 1.03 (0.90, 1.19) | 5145 | 1.23 (1.17, 1.29) |
| Per 100 g/d | |  | | 1.03 (1.01, 1.04) |  | 1.02 (0.99, 1.04) |  | 1.01 (0.99, 1.04) |  | 1.04 (1.03, 1.05) |
| Whole milk | | |  |  |  |  |  |  |  |  |
| Non-consumers | 0 | 568,415 | 1524 | 1 (ref.) | 727 | 1 (ref.) | 521 | 1 (ref.) | 4893 | 1 (ref.) |
| Q1 | 122 (91–147) | 472,172 | 1945 | 0.97 (0.91, 1.04) | 968 | 1.02 (0.92, 1.12) | 676 | 0.99 (0.88, 1.12) | 5791 | 0.97 (0.93, 1.01) |
| Q2 | 220 (196–245) | 426,771 | 1670 | 1.01 (0.94, 1.09) | 836 | 1.04 (0.94, 1.16) | 562 | 0.98 (0.86, 1.11) | 5003 | 1.00 (0.96, 1.05) |
| Q3 | 342 (305–382) | 405,739 | 1672 | 1.04 (0.96, 1.12) | 855 | 1.07 (0.96, 1.19) | 603 | 1.05 (0.92, 1.19) | 5128 | 1.07 (1.03, 1.12) |
| Q4 | 534 (471–626) | 374,059 | 1401 | 1.07 (0.98, 1.16) | 771 | 1.13 (1.01, 1.27) | 545 | 1.11 (0.96, 1.27) | 4532 | 1.16 (1.11, 1.22) |
| Per 100 g/d | |  | | 1.01 (1.00, 1.03) |  | 1.02 (1.00, 1.03) |  | 1.01 (0.99, 1.03) |  | 1.03 (1.02, 1.04) |
| Low-fat milk | |  |  |  |  |  |  |  |  |  |
| Non-consumers | 0 | 1,009,404 | 3669 | 1 (ref.) | 1935 | 1 (ref.) | 1345 | 1 (ref.) | 11,581 | 1 (ref.) |
| Q1 | 81 (61–99) | 316,269 | 1377 | 0.96 (0.90, 1.02) | 681 | 0.95 (0.87, 1.04) | 473 | 0.96 (0.86, 1.07) | 4113 | 0.93 (0.90, 0.97) |
| Q2 | 161 (142–176) | 299,347 | 1072 | 0.95 (0.88, 1.01) | 508 | 0.89 (0.80, 0.98) | 355 | 0.90 (0.80, 1.02) | 3300 | 0.94 (0.90, 0.98) |
| Q3 | 252 (221–285) | 305,916 | 1117 | 0.95 (0.88, 1.02) | 548 | 0.90 (0.81, 0.99) | 391 | 0.94 (0.84, 1.06) | 3439 | 0.96 (0.92, 0.99) |
| Q4 | 432 (368–530) | 316,219 | 977 | 1.05 (0.98, 1.14) | 485 | 0.98 (0.88, 1.09) | 343 | 1.02 (0.90, 1.16) | 2914 | 0.99 (0.95, 1.03) |
| Per 100 g/d | |  | | 1.01 (0.99, 1.02) |  | 0.99 (0.97, 1.01) |  | 1.00 (0.97, 1.02) |  | 1.00 (0.99, 1.01) |

^1^ Cox proportional hazards regression stratified by birth cohort (<1930, 1930-34, 1935-39, 1940-44, 1945-49, 1950-54, and ≥1955) with age as timescale, adjusted for sex, total energy intake (kJ), BMI (kg/m2), smoking status, recreational and occupational physical activity, education level, marital status, and comorbidities, other dietary sources of saturated fats (cheese, red and processed meat, fat on bread, meat, and fish), intake of coffee, sweetened beverages, dietary fiber, and vitamin C. HRs for overall milk are also adjusted for type of milk. “Non-consumers” are participants not consuming the respective type of milk or any milk.
AMI, acute myocardial infarction; CVD, cardiovascular disease; HR, hazard ratio; IHD, ischemic heart disease; IQR, interquartile range.

## **Supplementary Table 16.** Hazard ratios (HRs) with 95% confidence intervals (CIs) of cardiovascular disease (CVD), ischemic heart disease (IHD), acute myocardial infarction (AMI), and all-cause mortality by milk intake, excluding participants with self-reported comorbidities (N = 70,025).

|  | Median (IQR), g/day | CVD mortality | | | IHD mortality | | AMI mortality | | All-cause mortality | |
| --- | --- | --- | --- | --- | --- | --- | --- | --- | --- | --- |
| Milk type |  | Person-years | Deaths, N | HR (95% CI)^1^ | Deaths, N | HR (95% CI)^1^ | Deaths, N | HR (95% CI)^1^ | Deaths, N | HR (95% CI)^1^ |
| Overall milk | | |  |  |  |  |  |  |  |  |
| Q1 | 136 (98–159) | 398,013 | 1185 | 1 (ref.) | 591 | 1 (ref.) | 420 | 1 (ref.) | 3904 | 1 (ref.) |
| Q2 | 222 (202–246) | 437,720 | 1261 | 0.96 (0.89, 1.03) | 576 | 0.87 (0.78, 0.97) | 402 | 0.90 (0.78, 1.04) | 4389 | 1.03 (0.99, 1.08) |
| Q3 | 323 (297–349) | 430,250 | 1455 | 1.07 (0.99, 1.15) | 678 | 0.99 (0.89, 1.09) | 477 | 0.99 (0.86, 1.14) | 4724 | 1.10 (1.06, 1.15) |
| Q4 | 424 (398-454) | 430,987 | 1356 | 1.08 (1.00, 1.17) | 652 | 1.01 (0.90, 1.12) | 466 | 1.02 (0.88, 1.18) | 4544 | 1.15 (1.11, 1.21) |
| Q5 | 576 (527–653) | 434,005 | 1297 | 1.12 (1.03, 1.21) | 661 | 1.05 (0.94, 1.18) | 470 | 1.06 (0.91, 1.24) | 4481 | 1.24 (1.19, 1.30) |
| Per 100 g/d | |  | | 1.03 (1.02, 1.05) |  | 1.02 (1.00, 1.04) |  | 1.02 (0.99, 1.04) |  | 1.05 (1.04, 1.05) |
| Whole milk | | |  |  |  |  |  |  |  |  |
| Non-consumers | 0 | 544,183 | 1163 | 1 (ref.) | 508 | 1 (ref.) | 371 | 1 (ref.) | 4181 | 1 (ref.) |
| Q1 | 123 (92–148) | 436,284 | 1485 | 0.96 (0.89, 1.04) | 699 | 1.03 (0.92, 1.16) | 504 | 1.02 (0.89, 1.18) | 429 | 0.96 (0.92, 1.01) |
| Q2 | 220 (197–245) | 401,039 | 1312 | 1.00 (0.92, 1.09) | 619 | 1.06 (0.94, 1.20) | 418 | 0.98 (0.85, 1.14) | 4289 | 1.00 (0.95, 1.04) |
| Q3 | 342 (305–382) | 386,473 | 1412 | 1.06 (0.97, 1.16) | 688 | 1.11 (0.98, 1.26) | 488 | 1.09 (0.94, 1.26) | 4596 | 1.08 (1.03, 1.13) |
| Q4 | 534 (471–626) | 362,995 | 1182 | 1.05 (0.96, 1.16) | 644 | 1.18 (1.03, 1.35) | 454 | 1.14 (0.97, 1.33) | 4147 | 1.17 (1.11, 1.22) |
| Per 100 g/d | |  | | 1.01 (1.00, 1.03) |  | 1.02 (1.00, 1.04) |  | 1.01 (0.99, 1.04) |  | 1.03 (1.02, 1.04) |
| Low-fat milk | | |  |  |  |  |  |  |  |  |
| Non-consumers | 0 | 975,786 | 3166 | 1 (ref.) | 1629 | 1 (ref.) | 1132 | 1 (ref.) | 10,641 | 1 (ref.) |
| Q1 | 81 (61–99) | 289,898 | 1042 | 0.94 (0.87, 1.01) | 480 | 0.91 (0.82, 1.02) | 346 | 0.95 (0.84, 1.07) | 3415 | 0.92 (88, 0.96) |
| Q2 | 161 (142–176) | 278,844 | 793 | 0.93 (0.86, 1.01) | 344 | 0.85 (0.75, 0.96) | 237 | 0.84 (0.73, 0.97) | 279 | 0.93 (0.89, 0.97) |
| Q3 | 253 (221–285) | 284,488 | 832 | 0.97 (0.89, 1.05) | 379 | 0.91 (0.81, 1.03) | 278 | 0.97 (0.84, 1.11) | 2847 | 0.96 (0.92, 1.00) |
| Q4 | 433 (369–531) | 301,958 | 721 | 1.02 (0.94, 1.11) | 326 | 0.92 (0.81, 1.05) | 242 | 0.99 (0.85, 1.14) | 2410 | 0.96 (0.92, 1.01) |
| Per 100 g/d | |  | | 1.00 (0.99, 1.02) |  | 0.98 (0.96, 1.00) |  | 0.99 (0.96, 1.02) |  | 0.99 (0.98, 1.00) |

^1^ Cox proportional hazards regression stratified by birth cohort (<1930, 1930-34, 1935-39, 1940-44, 1945-49, 1950-54, and ≥1955) with age as timescale, adjusted for sex, total energy intake (kJ), BMI (kg/m2), smoking status, recreational and occupational physical activity, education level, marital status, and comorbidities, other dietary sources of saturated fats (cheese, red and processed meat, fat on bread, meat, and fish), intake of coffee, sweetened beverages, dietary fiber, and vitamin C. HRs for overall milk are also adjusted for type of milk. “Non-consumers” are participants not consuming the respective type of milk or any milk.
AMI, acute myocardial infarction; CVD, cardiovascular disease; HR, hazard ratio; IHD, ischemic heart disease; IQR, interquartile range.

## **Supplementary Table 17.** Hazard ratios (HRs) with 95% confidence intervals (CIs) of cardiovascular disease (CVD), ischemic heart disease (IHD), acute myocardial infarction (AMI), and all-cause mortality by milk intake, additionally adjusted for intake of bread, jam, syrup/honey, mayonnaise-based salads, chocolate spreads, and fish as bread toppings (e.g., herring, sardines), eggs, and beer/wine/liquor (n = 72,977).

|  | Median (IQR), g/day | CVD mortality | | | IHD mortality | | AMI mortality | | All-cause mortality | |
| --- | --- | --- | --- | --- | --- | --- | --- | --- | --- | --- |
| Milk type |  | Person-years | Deaths, N | HR (95% CI)^1^ | Deaths, N | HR (95% CI)^1^ | Deaths, N | HR (95% CI)^1^ | Deaths, N | HR (95% CI)^1^ |
| Overall milk | | |  |  |  |  |  |  |  |  |
| Q1 | 136 (97–159) | 424,486 | 1512 | 1 (ref.) | 785 | 1 (ref.) | 563 | 1 (ref.) | 4581 | 1 (ref.) |
| Q2 | 222 (202–247) | 469,277 | 1643 | 0.97 (0.90, 1.04) | 798 | 0.93 (0.83, 1.03) | 540 | 0.86 (0.76, 0.98) | 5212 | 1.03 (0.99, 1.08) |
| Q3 | 323 (297–349) | 464,315 | 1882 | 1.05 (0.97, 1.13) | 938 | 1.00 (0.90, 1.11) | 653 | 0.95 (0.84, 1.08) | 5642 | 1.09 (1.04, 1.14) |
| Q4 | 424 (398–454) | 461,543 | 1782 | 1.06 (0.98, 1.15) | 916 | 1.01 (0.90, 1.12) | 641 | 0.97 (0.85, 1.10) | 5408 | 1.12 (1.07, 1.18) |
| Q5 | 576 (527–653) | 460,047 | 1697 | 1.08 (1.00, 1.18) | 896 | 1.02 (0.91, 1.14) | 625 | 0.98 (0.85, 1.12) | 5239 | 1.19 (1.14, 1.25) |
| Per 100 g/d | |  |  | 1.02 (1.00, 1.03) |  | 1.00 (0.98–1.03) |  | 1.00 (0.98, 1.03) |  | 1.03 (1.03, 1.04) |
| Whole milk | | |  |  |  |  |  |  |  |  |
| Non-consumers | 0 | 578,246 | 1602 | 1 (ref.) | 774 | 1 (ref.) | 552 | 1 (ref.) | 5125 | 1 (ref.) |
| Q1 | 122 (91–147) | 480,098 | 2030 | 0.99 (0.92, 1.06) | 1013 | 1.03 (0.93, 1.13) | 707 | 1.01 (0.90, 1.13) | 5994 | 0.98 (0.94, 1.02) |
| Q2 | 220 (196–245) | 432,280 | 1727 | 1.02 (0.95, 1.09) | 870 | 1.05 (0.95, 1.16) | 583 | 0.98 (0.87, 1.11) | 5147 | 1.01 (0.97, 1.05) |
| Q3 | 342 (305–382) | 411,081 | 1729 | 1.04 (0.97, 1.12) | 888 | 1.06 (0.96, 1.18) | 626 | 1.05 (0.92, 1.19) | 5253 | 1.07 (1.02, 1.12) |
| Q4 | 534 (471–626) | 377,962 | 1428 | 1.05 (0.97, 1.15) | 788 | 1.12 (1.00, 1.26) | 554 | 1.09 (0.95, 1.25) | 4617 | 1.15 (1.10, 1.21) |
| Per 100 g/d | |  |  | 1.01 (1.00, 1.02) |  | 1.01 (0.99, 1.03) |  | 1.01 (0.98, 1.03) |  | 1.03 (1.02, 1.04) |
| Low-fat milk | |  |  |  |  |  |  |  |  |  |
| Non-consumers | 0 | 1,019,485 | 3760 | 1 (ref.) | 1990 | 1 (ref.) | 1380 | 1 (ref.) | 11,812 | 1 (ref.) |
| Q1 | 81 (61–99) | 322,039 | 1432 | 0.97 (0.91, 1.03) | 710 | 0.96 (0.88, 1.05) | 493 | 0.97 (0.87, 1.08) | 4252 | 0.94 (0.90, 0.97) |
| Q2 | 161 (142–176) | 303,985 | 1120 | 0.94 (0.88, 1.01) | 535 | 0.88 (0.79, 0.97) | 372 | 0.89 (0.79, 1.01) | 3429 | 0.94 (0.90, 0.98) |
| Q3 | 252 (221–285) | 312,319 | 1182 | 0.96 (0.89, 1.03) | 588 | 0.92 (0.83, 1.01) | 417 | 0.96 (0.85, 1.08) | 3610 | 0.96 (0.92, 1.00) |
| Q4 | 431 (368–530) | 321,838 | 1022 | 1.05 (0.97, 1.13) | 510 | 0.97 (0.87, 1.08) | 360 | 1.00 (0.89, 1.14) | 3033 | 0.98 (0.94, 1.03) |
| Per 100 g/d | |  |  | 1.01 (0.99, 1.02) |  | 0.99 (0.97, 1.01) |  | 0.99 (0.97, 1.02) |  | 1.00 (0.99, 1.01) |

^1^ Cox proportional hazards regression stratified by birth cohort (<1930, 1930-34, 1935-39, 1940-44, 1945-49, 1950-54, and ≥1955) with age as timescale, adjusted for sex, total energy intake (kJ), BMI (kg/m2), smoking status, recreational and occupational physical activity, education level, marital status, and comorbidities, other dietary sources of saturated fats (cheese, red and processed meat, fat on bread, meat, and fish), intake of coffee, sweetened beverages, dietary fiber, and vitamin C, bread, jam, syrup/honey, mayonnaise-based salads, chocolate spreads, and fish as bread toppings (e.g., herring, sardines), eggs, and beer/wine/liquor. HRs for overall milk are also adjusted for type of milk. “Non-consumers” are participants not consuming the respective type of milk or any milk.
AMI, acute myocardial infarction; CVD, cardiovascular disease; HR, hazard ratio; IHD, ischemic heart disease; IQR, interquartile range.
